# Advances and Perspectives in Gate Dielectric Thin Films for 4H-SiC MOSFETs

**DOI:** 10.3390/ma19040766

**Published:** 2026-02-15

**Authors:** Zhaopeng Bai, Jinsong Liang, Chengxi Ding, Zimo Zhou, Man Luo, Lin Gu, Hong-Ping Ma, Qing-Chun Zhang

**Affiliations:** 1 Institute of Wide Bandgap Semiconductors and Future Lighting, College of Intelligent Robotics and Advanced Manufacturing, Fudan University, Shanghai 200433, China; zpbai24@m.fudan.edu.cn (Z.B.); 25213070008@m.fudan.edu.cn (J.L.); 23210860003@m.fudan.edu.cn (C.D.); 24210860099@m.fudan.edu.cn (Z.Z.); 24210720123@m.fudan.edu.cn (M.L.); 24110860041@m.fudan.edu.cn (L.G.); 2 Shanghai Research Center for Silicon Carbide Power Devices Engineering & Technology, Fudan University, Shanghai 200433, China; 3 Institute of Wide Bandgap Semiconductor Materials and Devices, Research Institute of Fudan University in Ningbo, Ningbo 315327, China

**Keywords:** SiC MOSFET, SiO_2_, high-k, interfaces, ALD

## Abstract

The performance and reliability of 4H-SiC Metal-Oxide-Semiconductor Field-Effect Transistors (MOSFETs) are largely determined by the material properties of gate dielectric films and the quality of the dielectric/SiC interface. This paper provides a systematic review of recent progress in gate dielectric engineering for 4H-SiC MOSFETs, with emphasis on SiO_2_-based gate dielectrics and high-dielectric-constant (high-k) gate dielectrics. First, for conventional thermally grown SiO_2_/SiC systems, the effects of interface nitridation, gate oxide doping, and surface pretreatment techniques are comprehensively discussed. The influence mechanisms of these processes on carbon-related interface defects, interface state density and field-effect mobility are analyzed, and the advances in related research are summarized. Second, the application of high-k gate dielectrics, including Al_2_O_3_, HfO_2_, ZrO_2_, and stacked dielectric structures, in SiC MOS devices is systematically reviewed. The advantages of these materials in reducing equivalent oxide thickness, increasing gate capacitance, suppressing leakage current, and improving thermal stability are highlighted. In addition, interface defects and electrical characteristics associated with different high-k gate dielectrics are comparatively evaluated. Finally, future research directions are discussed, including in situ interface engineering based on atomic layer deposition, dopant modulation, and heterogeneous gate dielectric structures. These approaches show strong potential for achieving high mobility, low loss, and high reliability in advanced 4H-SiC power MOSFETs.

## 1. Introduction

With the rapid development of renewable energy generation, electric vehicles, high-speed rail transportation, and smart grids, power electronic systems are evolving toward higher power density, higher efficiency, and higher operating temperature. Conventional silicon (Si)-based power devices are approaching the fundamental material limits in breakdown voltage, switching loss, and high-temperature reliability, and therefore cannot satisfy the requirements of next-generation high-performance power electronic systems [1,2]. In this context, silicon carbide (SiC) has attracted increasing attention due to its superior physical properties. SiC exhibits a wide bandgap of about 3.26 eV, a high critical electric field nearly ten times higher than that of Si, high thermal conductivity, and high electron saturation drift velocity. These advantages enable SiC devices to demonstrate significantly improved performance under high-voltage, high-frequency, and high-temperature operating conditions compared with Si-based devices [3,4]. Among various SiC power devices, SiC power Metal-Oxide-Semiconductor Field-Effect Transistors (MOSFETs) have emerged as one of the most promising core devices for medium- and high-voltage power electronics in the range of 600 V to 10 kV. Voltage-controlled operation, low on-state loss, and excellent switching characteristics have promoted the gradual commercialization of SiC MOSFET technology in recent years [5,6].

**Figure 1:**
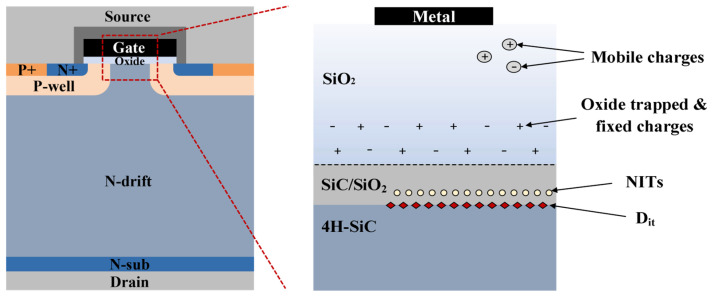
Oxide traps, interface defect charges, and near-interface trap locations at the SiO_2_/4H-SiC interface.

SiC offers a distinct advantage for power device applications due to its ability to form silicon oxide (SiO_2_) through direct thermal oxidation, which can serve as the gate dielectric in MOS structures. However, unlike mature Si technology, SiC MOS technology faces fundamental challenges related to interface quality. These issues stem mainly from the formation of defects such as carbon clusters during the thermal oxidation of SiC, which introduce a high density of trapped charges at the SiC/SiO_2_ interfaces [7,8,9,10,11]. As illustrated in Figure 1, the dominant defect types at this interface can be classified into several categories: interface state density (*D*_it_) near the SiC/SiO_2_ boundary, near-interface oxide traps (NITs), oxide trapped charges, fixed oxide charges (*Q*_ox_), and mobile ions.

Interface traps are localized states situated directly at the oxide/semiconductor interface. They primarily originate from lattice mismatch, broken chemical bonds, and carbon-related byproducts generated during thermal oxidation [12]. Distributed across the SiC bandgap, their density typically peaks near the conduction and valence band edges. *D_it_* degrades channel carrier transport and recombination, reduces the concentration of free carriers, enhances interface scattering, and consequently lowers the field-effect mobility (*µ*_fe_) while increasing the specific on-resistance [13,14].

**Figure 2:**
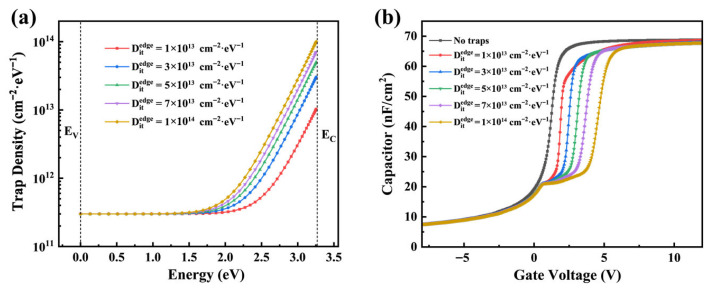
(**a**) Energy distribution of *D_it_*. (**b**) Influence of different *D_it_* levels on the C–V characteristics of the SiC MOS capacitor.

In practical MOS capacitors, interface traps within the SiC bandgap usually exhibit an approximately U-shaped distribution. This distribution can be approximated by a superposition of a uniform distribution and an exponential distribution. To provide an intuitive illustration of the physical impact of *D_it_* and NITs on the C–V characteristics of SiC MOS capacitors, TCAD-based numerical simulations were employed as a tool for elucidating qualitative trends. The simulations were performed for n-type 4H-SiC MOS capacitors using Sentaurus TCAD within a drift–diffusion framework. Carrier transport was modeled using a combination of the Arora, Canali, and Lombardi mobility models to account for bulk mobility, high-field effects, and interface scattering, respectively [15]. Gate oxide current mechanisms included direct tunneling, Fowler–Nordheim tunneling, and trap-assisted tunneling, while carrier recombination was described using the Shockley–Read–Hall model [16,17]. Interface-related defects were represented by acceptor–type interface states and near-interface neutral electron traps, enabling a qualitative analysis of the influence of *D_it_* and NITs on the evolution of C–V characteristics. TACD simulations are employed to investigate the energy level distribution of the introduced *D_it_*, as shown in Figure 2a. Figure 2b compares the C–V characteristics with different introduced *D_it_* values and the ideal defect-free condition. The results clearly indicate that a higher *D_it_* leads to a more pronounced positive shift in the C–V curve, while the slope of the curve decreases accordingly. In addition, after introducing a high density of deep-level traps, a short plateau appears in the depletion region of the C–V curve.

NITs are located within the oxide layer, about 1–2 nm from the SiC interface. They are mainly associated with oxygen vacancy-related defects in the oxide, while localized defect states such as Si–O–C complexes formed by carbon diffusion during oxidation can also contribute [12]. NITs notably affect the threshold voltage (*V*th) stability of devices. NITs exhibit slow charge capture and emission, which induces a sweep-direction dependence of the C–V characteristics. The energy level position and concentration of NITs govern the electron capture rate, leading to different hysteresis magnitudes between forward and reverse C–V sweeps.

**Figure 3:**
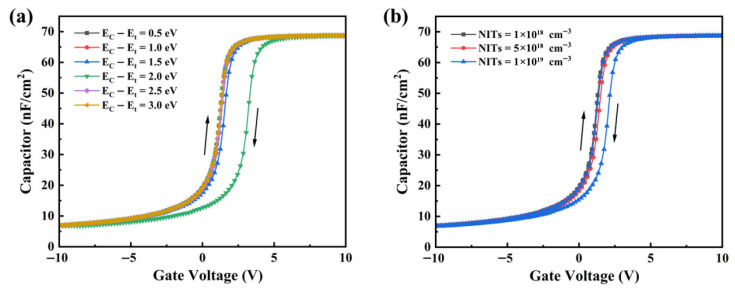
Influence of near-interface defects on C–V characteristics: (**a**) effect of defect energy level position and (**b**) effect of defect concentration.

In the simulation, NITs are placed in the oxide within 2 nm from the interface and follow a single-level distribution. Figure 3a compares the C–V curves of NITs with a trap density of 1 × 10^18^ cm^−3^ and a single-level distribution at different energy levels with the ideal defect-free C–V curve. As the trap level moves farther away from the SiO_2_ conduction band edge, its separation from the SiC conduction band edge first decreases and then increases. A smaller separation from the SiC conduction band edge enables more electrons to be captured, resulting in an increase followed by a decrease in the hysteresis voltage. The hysteresis voltage reaches the maximum when the trap level aligns with the SiC conduction band edge. Figure 3b shows the C–V analysis for different near-interface trap densities. The hysteresis becomes progressively more pronounced with increasing near-interface trap density because a higher trap density captures more electrons.

Fixed oxide charges (*Q*_ox_) arise from deep-level dangling bonds, carbon clusters, and suboxide species that do not form a complete lattice during thermal oxidation [18]. These charges are immobile and cannot be charged or discharged under varying surface potentials; they are generally positive, attract electrons in the semiconductor, shift the flatband condition, and thereby increase the *V*th. Oxide trapped charges result from intrinsic oxidation-induced defects within, and from residual carbon-related species (e.g., CO). These traps can become activated at elevated temperatures, degrading *V*th stability and gate oxide reliability. Mobile charges are mainly caused by alkali metal contamination during the oxidation process. They exist predominantly as K^+^ and Na^+^ ions in the gate oxide. Under high-temperature and high-electric-field conditions, these ions migrate, leading to drift in the *V*th.

**Figure 4:**
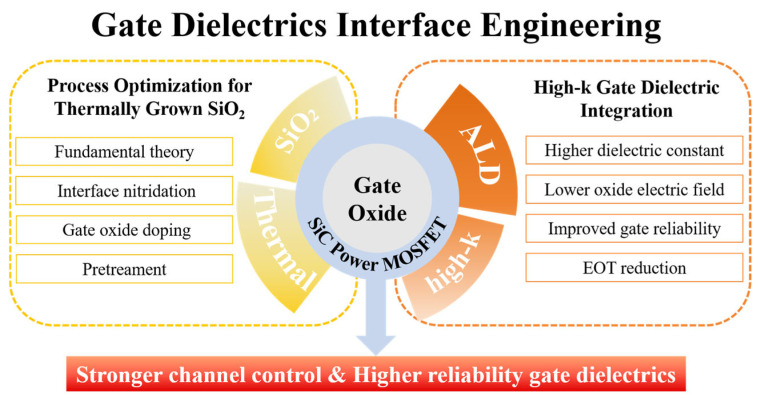
Gate oxide engineering for SiC power MOSFET via thermally grown SiO_2_ interface optimization and ALD high-k integration.

Therefore, gate dielectric engineering plays a central role in the research and development of SiC MOSFETs, as shown in Figure 4 [19]. On the one hand, process optimization is required to improve the interface quality of conventional thermally grown SiO_2_ gate dielectrics. On the other hand, the introduction of high-dielectric-constant gate dielectrics has been extensively investigated to reduce the equivalent oxide thickness (EOT) and the electric field across the gate oxide, enhance channel electrostatic control, mitigate the influence of *D_it_*, and improve gate oxide reliability. These topics have become major research focuses in the field of SiC MOSFETs. A systematic summary and analysis of these issues are of great significance for a deeper understanding of the physical mechanisms of SiC MOSFETs and for further improvement of device performance and reliability.

The diverse defects present at the SiC/SiO_2_ interface formed via conventional thermal oxidation severely restrict the physical and electrical characterizations of practical SiC MOSFETs. To address this challenge, several interface optimization strategies compatible with thermal oxidation processes have been developed. These primarily include interface nitridation through annealing in N-containing atmospheres, gate oxide modification by doping with specific elements, and surface pretreatment of the substrate prior to oxidation.

Although significant improvements in interface quality and device performance have been achieved for SiO_2_ gate dielectrics in SiC MOSFETs through the optimization of thermal oxidation conditions and post-oxidation annealing processes, critical limitations remain in fully exploiting the material potential of SiC. On the one hand, carbon-related defects and NITs at the SiC and SiO_2_ interface cannot be completely eliminated. As a result, *D_it_* remains at a relatively high level, and channel carrier mobility is significantly lower than that of Si MOSFETs. On the other hand, as SiC MOSFETs evolve toward higher power density, smaller cell dimensions, and higher operating electric fields, conventional SiO_2_ gate dielectrics increasingly exhibit limitations in EOT scaling, gate controllability enhancement, and electric field distribution optimization. These limitations originate from the intrinsically low dielectric constant of SiO_2_ [20].

In this context, high-k gate dielectric materials have been proposed as an important approach to overcome the limitations of conventional SiO_2_ gate oxide systems. By increasing the dielectric constant of the gate dielectric, high-k materials enable a significant reduction in EOT while maintaining a relatively large physical thickness. This property enhances the electric field control of the gate over the channel while suppressing gate leakage current. As a result, high-k gate dielectrics exhibit potential advantages in mitigating short channel effects, optimizing channel carrier distribution, and reducing interface scattering effects [21]. In recent years, various high-k materials, including HfO_2_, ZrO_2_, Al_2_O_3_, AlN, and rare-earth oxides, have been introduced into gate dielectric research for SiC MOSFETs [22,23,24,25,26,27]. In several experimental studies, these materials have demonstrated electrical characteristics superior to those of conventional SiO_2_ gate dielectrics. Therefore, a systematic understanding of the material and physical requirements of high-k gate dielectrics for SiC MOSFETs is a critical foundation for sustained progress in this field.

A primary objective of introducing high-k gate dielectrics into Si MOSFETs is to achieve effective scaling of EOT while maintaining a relatively large physical thickness, thereby reducing gate leakage current and improving dielectric reliability. However, in SiC MOSFETs, the significance of EOT scaling extends beyond enhanced gate electrostatic control and is closely related to the regulation of high-electric-field distribution. SiC devices typically operate under high-voltage conditions, and the gate dielectric region is therefore subjected to higher local electric field stress. According to Gauss’s law, the electric field distribution within the gate dielectric is strongly influenced by the dielectric constant of the material [25]:

(1) $$E_{\mathrm{SiC}}\kappa_{\mathrm{SiC}}\;=\;E_{\mathrm{oxide}}\kappa_{\mathrm{oxide}}$$

where *E_SiC_* denotes the electric field in the SiC epitaxial layer, and *κ_SiC_* represents the dielectric constant of SiC. *E_oxide_* denotes the electric field in the gate dielectric, and *κ_oxide_* represents the dielectric constant of the gate dielectric. The dielectric constant of 4H-SiC is approximately 2.5 times that of SiO_2_. As a result, in a SiO_2_ and 4H-SiC structure, the electric field sustained by the SiO_2_ gate oxide is about 2.5 times higher than that in 4H-SiC. During the gradual increase in the applied electric field, the SiO_2_ layer is therefore more likely to experience dielectric breakdown first. Consequently, the critical breakdown electric field of devices containing a SiO_2_ and 4H-SiC interface is significantly lower than the intrinsic critical breakdown field of 4H-SiC. This result indicates that, in conventional SiO_2_ and 4H-SiC systems, the high breakdown field advantage of 4H-SiC cannot be fully utilized due to the relatively low dielectric constant and breakdown strength of SiO_2_. If dielectric nonuniformity or defect clustering exists within high-k materials, local electric field enhancement effects will be significantly intensified. This behavior increases the risk of dielectric breakdown and reliability degradation. Therefore, the requirements imposed by SiC MOSFETs on high-k gate dielectrics extend beyond a high dielectric constant. Greater emphasis is placed on spatial uniformity of the dielectric constant, material density, and stability under high electrics.

In SiC MOSFETs, the band alignment of high-k gate dielectrics directly determines gate leakage mechanisms and reliability behavior. Owing to the wide bandgap of 4H-SiC of about 3.26 eV, the conduction band minimum of SiC is located at a relatively high energy level. In contrast, the conduction band minimum of many commonly used high-k oxides is not sufficiently high, resulting in an inadequate conduction band offset. When the conduction band offset is small, channel electrons can more easily enter the high-k gate dielectric through thermally activated emission, tunneling, or trap-assisted injection under high-electric-field or high-temperature conditions. This process leads to increased gate leakage current and triggers charge trapping, *V*th drift, and bias temperature instability. These effects collectively degrade device reliability [28]. Therefore, from a material selection perspective, high-k gate dielectrics for SiC MOSFETs must achieve a proper balance between dielectric constant and band structure rather than pursuing a higher dielectric constant alone.

The fabrication and operating environments of SiC MOSFETs require gate dielectrics with excellent thermal stability. During device fabrication, processes such as annealing and metal contact formation impose thermal stress on gate dielectric materials. In practical applications, many SiC MOSFETs operate under long-term high-temperature conditions. Some high-k oxides are prone to crystallization, phase transformation, or interfacial reactions at elevated temperatures. These phenomena can significantly degrade *D_it_* and electrical performance. Therefore, from a material perspective, high-k gate dielectrics must not only exhibit good initial properties after deposition but also maintain structural and chemical stability under high-temperature stress. This requirement has motivated extensive efforts to improve the thermal stability of high-k gate dielectric systems through material doping, the introduction of interfacial layers, and the optimization of annealing processes [26,29,30].

Reliability issues of high-k gate dielectrics in SiC MOSFETs are fundamentally associated with material defects and defect dynamics. Oxygen vacancies, impurity atoms, and structurally disordered regions are inevitably present in high-k films and actively participate in charge trapping and detrapping processes under high-electric-field and high-temperature conditions. These processes directly lead to bias temperature instability, *V*th drift, and time-dependent degradation of device parameters [31]. Therefore, from material and physical perspectives, the ultimate requirement for high-k gate dielectrics in SiC MOSFETs is to minimize intrinsic defect density and interface defect activity while maintaining adequate dielectric performance. In addition, defect behavior must remain controllable and predictable under electrical and thermal stress. This requirement explains the strong coupling between material design and interface engineering in current high-k gate dielectric research.

In summary, SiC power MOSFETs are representative power devices and exhibit significant advantages in high-voltage, high-frequency, and high-temperature applications. Device performance and reliability are largely constrained by gate dielectric properties and the quality of the dielectric and SiC interface. Thermally grown SiO_2_ gate dielectrics remain the mainstream choice for SiC MOSFETs because of high process maturity and good thermal stability. Intrinsic interface defects, however, limit further improvement of *µ*_fe_ and overall device performance. High-k gate dielectrics and stacked dielectric structures provide alternative approaches to enhance gate controllability and reduce EOT and gate oxide electric field strength. These approaches still face challenges related to interface quality, thermal stability, and long-term reliability.

Therefore, two core technical routes have been established in current gate dielectric research for SiC MOSFETs. One route focuses on continuous optimization of SiO_2_ gate dielectrics. The other route emphasizes the exploration of high-k gate dielectrics and related interface engineering. To systematically summarize research progress, key challenges, and future trends in this field, this paper first provides a detailed review of the formation mechanisms of thermally grown SiO_2_ gate dielectrics in SiC MOSFETs. Interface control methods and associated impacts on device performance are also discussed. The paper then focuses on the current status, advantages, and challenges of high-k gate dielectrics and stacked gate structures. Finally, future directions of gate dielectric engineering for SiC MOSFETs are outlined. For clarity, this review primarily focuses on gate dielectric materials, interface physics, defect mechanisms, and their impact on the electrical performance and reliability of SiC MOS devices. Although system-level aspects such as device packaging, application-oriented circuit design, compact modeling, and industrial qualification standards are essential for the practical deployment of SiC power devices [32,33,34], they are beyond the scope of the present review and are therefore not discussed in detail.

## 2. Thermal Oxidation

**Figure 5:**
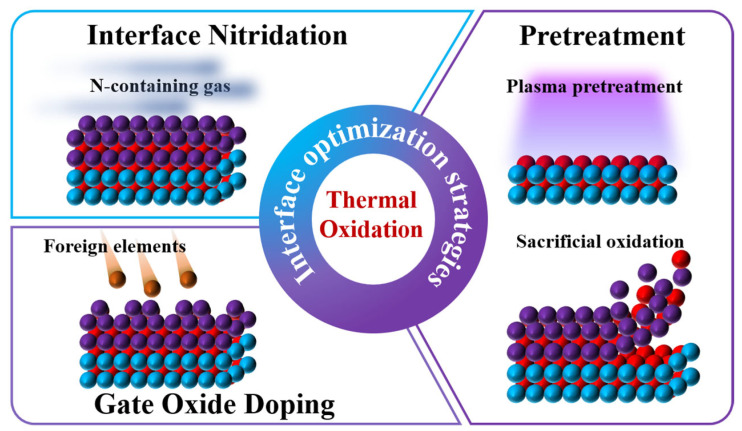
Schematic illustration of interface optimization strategies for thermally oxidized gate dielectrics in 4H-SiC MOSFETs.

SiC holds a distinctive position in power semiconductor applications owing to its ability to form high-quality SiO_2_ through direct thermal oxidation. However, this oxidation process inevitably introduces carbon-related defects at the SiO_2_/SiC interface. These defects result in a high *D_it_* and NITs, which severely degrades the *µ*_fe_ and thus poses a major challenge for SiC power MOSFET development. To address this challenge, several interface optimization strategies applicable in oxidation processes have been developed. These primarily include interface nitridation via annealing in nitrogen-containing atmospheres, doping modification of the gate oxide with selected elements, and surface pretreatment techniques before oxide deposition, as shown in Figure 5. A systematic understanding of the mechanisms and recent advances in these interface optimization techniques is essential for improving interface quality and realizing high-performance SiC MOSFETs. The following sections provide a detailed discussion.

### 2.1. Fundamental Aspects of SiC Oxidation

Thermally grown SiO_2_ is often used as the gate dielectric in MOS devices, as well as a passivation layer on the material surface. In commercial SiC power MOSFETs, the standard gate stack formation typically involves dry oxidation followed by interface nitridation [2,11]. Regardless of the gate stack structure and fabrication process, the SiO_2_/SiC interface fundamentally constitutes the oxidation front. Therefore, a thorough grasp of the fundamental aspects of SiC thermal oxidation serves as an essential prerequisite for understanding subsequent interface optimization strategies.

The general expression of SiC oxidation can be separated into the following reaction:

2SiC(s) + 3O_2_(g) → 2SiO_2_(s) + 2CO(g) ↑ (2)

The equation indicates that the product of the thermal oxidation of SiC is SiO_2_, which has been verified by various physical characterization techniques, such as X-ray photoelectron spectroscopy (XPS), auger electron spectroscopy (AES) and electron energy loss spectroscopy (EELS) [35,36,37]. By considering the atomic density of Si in the SiC crystal, it can be calculated that the consumption of Si during SiC thermal oxidation is about 46%, which is close to the value for thermal oxidation of pure Si. This implies that growing a 100 nm thick oxide layer would consume approximately 46 nm of the SiC material.

Early studies on the oxidation kinetics of SiC employed the Deal–Grove model originally developed for Si processing [38]:

(3) $$d_{\;\mathrm{ox}}^{\;2}\;+\;Ad_{\mathrm{ox}}\;=\;\mathrm{Bt}$$

where *d*_ox_ represents the oxide thickness, *t* is the oxidation time, *B* denotes the parabolic rate constant, and *B/A* corresponds to the linear rate constant. The Deal–Grove model accounts for both the initial reaction-controlled regime and the subsequent diffusion-controlled regime of the oxidation process. In the early stage of oxidation, when the oxide layer is thin, the oxidation rate is primarily limited by the chemical reaction at the interface, falling within the surface-reaction-controlled regime, where oxide thickness grows linearly with time. As the oxide thickens, the diffusion of oxygen through the growing oxide gradually becomes the rate limiting step, causing the oxidation rate to decrease. In this regime, the oxide thickness becomes proportional to the square root of oxidation time. However, with the progress of research, it has been found that, when the Deal–Grove model is applied to describe the oxidation process of SiC [39,40], the calculated results show poor agreement with experimental data, especially for oxide thicknesses below 20 nm. In SiC, the oxide growth rates on both the C-face and Si-face are significantly higher than those predicted by the Deal–Grove model. Analysis reveals two main reasons for this discrepancy. On the one hand, the Deal–Grove model, originally established for Si oxidation, does not account for the influence of gaseous reaction products on the oxidation kinetics, an effect that cannot be neglected in the case of SiC oxidation. On the other hand, the dependence of oxidation rate on crystal orientation is much stronger in SiC than in Si [41,42]. Specifically, the (000-1) plane (C-face) consistently exhibits a lower oxidation rate than the (0001) plane (Si-face), while the (11–20) and (1–100) planes show intermediate oxidation rates. Therefore, to accurately describe the oxidation behavior of different SiC crystal faces, the Deal–Grove model requires further modification or new oxidation kinetic models need to be developed [40,43,44].

**Figure 6:**
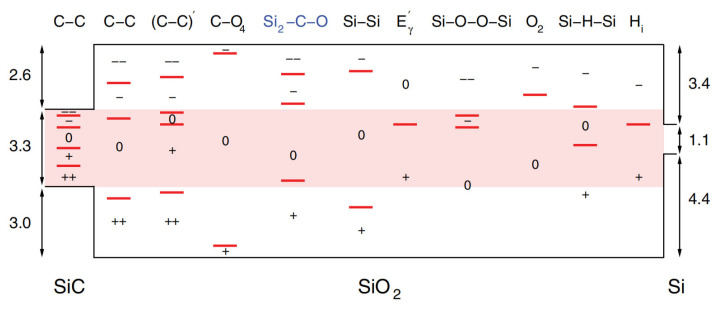
Schematic diagram of the charge transition levels of different defects at the SiO_2_/SiC interface [48].

To develop a fundamental understanding of SiO_2_ growth on SiC distinct from silicon oxidation, researchers are actively investigating this process. As SiC is a compound material composed of Si and C atoms, the role of C atoms during the thermal growth process is particularly critical. During the thermal oxidation of SiC, C atoms primarily escape the oxide layer as gaseous CO, though a small fraction may also diffuse into the substrate or accumulate near the interface. Numerous studies have been carried out to characterize and theoretically model the structure, composition distribution, and defect formation processes at the SiC/SiO_2_ interface [45,46,47]. Early work by Devynck et al. based on hybrid density functional theory, revealed that the defect system at the SiC/SiO_2_ interface is complex, involving carbon clusters as well as various chemical bonds such as C–C, C–O_4_, Si_2_–C–O, Si–Si, and Si–O–O–Si [48], as shown in Figure 6. Zhang et al. later investigated the origin of interface states by analyzing the chemical potentials of C and O at the interface and they demonstrated that only carbon clusters possess a sufficiently low formation energy (0.29 eV/C) to create a high density of defect states within the SiO_2_ layer, leading to carrier trapping and mobility degradation [49]. More recently, combining first principles and thermodynamic analysis, Wei et al. revealed that carbon-related point defects critically influence oxygen adsorption sites and energies, resulting in the formation of various defects during oxidation, such as carbon clusters, Si-dangling bonds, and Si–O–C bonds [50]. These defects can introduce interface states that directly affect the stability and reliability of SiC MOS devices [51].

### 2.2. Interface Optimization Strategies Applicable in Thermal Oxidation

Over the past few decades, a substantial number of papers have been published on SiC MOS structures. Despite continuous improvement in the quality of SiO_2_/SiC interfaces, their performance remains far from ideal. Therefore, developing effective interface strategies to reduce both *D*_it_ and NITs is crucial for advancing high-quality SiC MOS structures and high-performance 4H-SiC MOSFETs. This subsection begins by outlining interface nitridation techniques via annealing in N-containing atmospheres. It then discusses strategies for modifying the SiO_2_/SiC interfaces through the incorporation of foreign elements and examines their impact on device characteristics. Finally, novel pretreatment and sacrificial oxidation methods aimed at achieving a clean, contamination-free SiO_2_/SiC interface are reviewed.

#### 2.2.1. Interface Nitridation

**Figure 7:**
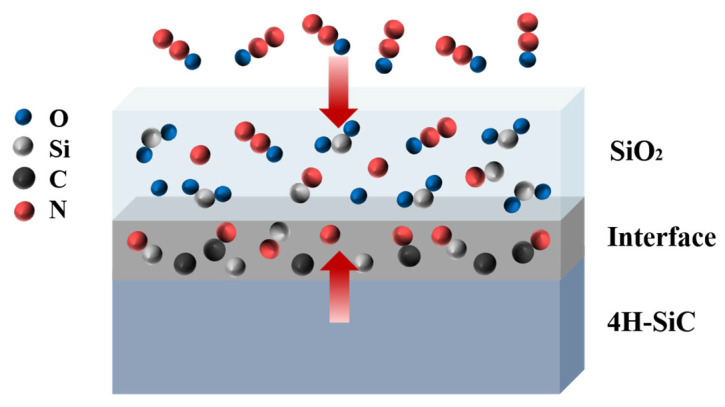
Schematic diagram illustrating the nitridation at SiC/SiO_2_ interface.

One widely used way to improve the properties of SiC MOS structures is post-oxidation annealing (POA) in a nitrogen-containing gas such as nitric oxide (NO) [52,53,54,55,56,57], nitrous oxide (N_2_O) [58,59,60], or ammonia (NH_3_) [61]. Direct oxidation in N_2_O or NO atmospheres was also proposed. In particular, interface nitridation by NO or N_2_O is widely adopted both in academic research and in the mass production of commercial SiC power MOSFETs. Regarding the possible passivation mechanisms and atomic structure of the nitrided SiO_2_/SiC interface, studies have shown that a large amount of nitrogen remains at the interface even after complete oxide removal [60,61]. The areal density of nitrogen atoms at the interface is strongly dependent on the nitridation conditions and can reach levels of 5 × 10^20^ cm^−3^ or higher. Consequently, the incorporation of nitrogen into the SiO_2_/SiC interface not only promotes the passivation of Si-dangling bonds into Si-N bonds, but also improves the removal of carbon-related defects through the formation of C–N bonds, as illustrated in Figure 7. This combined mechanism effectively reduces the *D*_it_ and enhances the *µ*_fe_ [62].

Early studies on nitridation treatments indicated that NO annealing exerts a beneficial effect on the SiO_2_/6H-SiC interface [52,53]. Subsequent investigations further confirmed the efficacy of NO annealing at the SiO_2_/4H-SiC interface [55], demonstrating that nitrogen accumulates only at the interface region, therefore reducing the *D*_it_ by approximately one order of magnitude. Owing to the high toxicity of NO, N_2_O nitridation has been proposed as a potential alternative [58,59,60]. However, the inferior performance of N_2_O annealing compared to NO annealing is primarily attributed to its thermal decomposition behavior at elevated temperatures (≈1200 °C), where N_2_O dissociates into a small fraction of NO along with substantial amounts of N_2_ and O_2_ [63]. These byproducts initiate competing reactions that impede the efficient incorporation of nitrogen into the SiO_2_/SiC interface, thereby diminishing the overall process effectiveness.

A previous study has reported the distribution of *D*_it_ near the conduction and valence band edges in n-type and p-type 4H-SiC (0001) MOS capacitors [64]. It compares interfaces formed by dry oxidation with those subjected to post-oxidation annealing in NO or N_2_O ambient, with the *D*_it_ value evaluated by the high (1 MHz)–low method. A clear reduction in the *D*_it_ across the entire bandgap energy range is observed after nitridation annealing. As a result, the *µ*_fe_ of n-channel of n-channel 4H-SiC (0001) MOSFETs fabricated on lightly doped p-type epilayers increases from 4 to 8 cm^2^·V^−1^·s^−1^ for dry oxidized samples to 40–52 cm^2^·V^−1^·s^−1^ after NO annealing [56,57] and to 25–35 cm^2^·V^−1^·s^−1^ after N_2_O annealing [58,59].

Since the initial proposal of interface nitridation, NO and N_2_O gases have been widely employed, with interfacial nitrogen concentration typically controlled by adjusting annealing time and temperature. However, interface nitridation remains insufficient to meet the requirements of high-performance SiC MOSFETs. This limitation stems from two primary factors. First, during annealing in oxygen-containing NO and N_2_O atmospheres, oxidation of the SiC surface proceeds simultaneously, making it difficult to precisely control the nitrogen concentration and distribution near the oxidation front. As a result, the optimal nitrogen concentration for the SiO_2_/4H-SiC interface remains unclear as yet. Second, the typical POA temperature range in nitrogen-containing atmospheres lies between 1000 °C and 1500 °C [65]. While elevated temperature promotes the nitridation process, it also increase the risk of introducing additional carbon clusters and silicon-dangling bonds due to the instability of the SiC surface at high temperatures. Concurrent re-oxidation may also occur, potentially generating new interface states [65,66].

**Figure 8:**
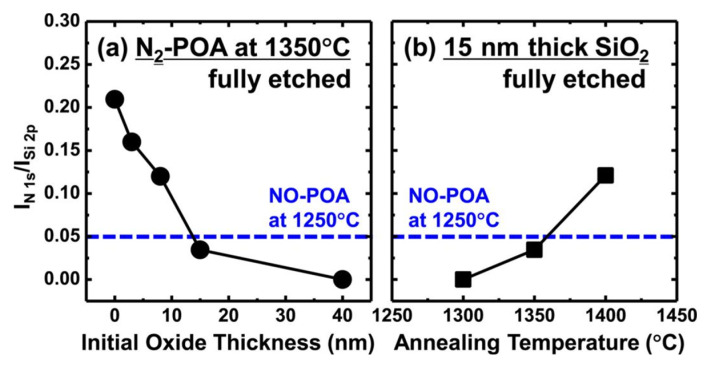
Nitrogen incorporation at the SiO_2_/SiC interface via high-temperature N_2_ annealing. The ratio of N 1s to Si 2p XPS peak intensities (I_N1s_/I_Si2p_) is plotted versus (**a**) initial oxide thickness and (**b**) annealing temperature [67].

To address these limitations, alternative nitridation methods have been explored in recent years. One approach utilizes pure nitrogen gas (N_2_), where high-temperature annealing on a bare SiC surface forms a thin SiON layer. Despite the relatively low reactivity of N_2_ molecules, annealing at 1350 °C enables effective interfacial nitridation and completely suppresses additional oxide growth. The nitrogen concentration mainly depends on the initial oxide thickness and the annealing temperature, as shown in Figure 8a,b [67,68]. Notably, this method can introduce a higher nitrogen dose under harmless conditions compared to conventional NO treatments. Although pure N_2_ annealing imposes relatively restrictive process conditions (high temperature and thin oxides), subsequent studies extended its utility by combining chemical vapor deposition (CVD)-grown SiO_2_ films with low-temperature oxygen pre-annealing prior to N_2_ treatment [69]. SiC MOSFETs fabricated with 40–45 nm thick CVD-SiO_2_ gate dielectrics and N_2_ annealing at 1300 °C exhibited a lower *D*_it_ (4 × 10^11^ eV^−1^·cm^−2^ at *E*_C_ − *E* = 0.2 eV) and a higher *µ*_fe_ (50 cm^2^·V^−1^·s^−1^ at *N*_A_ = 1 × 10^15^ cm^−3^) compared to devices with conventional NO processing.

Another emerging method involves low-temperature processing using supercritical fluids. Wang et al. [70] employed supercritical N_2_O fluid (SCN_2_O) treatment to improve SiO_2_/4H-SiC interfacial quality. After SCN_2_O post-oxidation annealing, the interface state density was significantly reduced to 2.8 × 10^11^ eV^−1^·cm^−2^, while the maximum oxide critical electric field increased by 18.19%. Additionally, NITs were reduced by 69.90% compared to those after N_2_O POA. Critically, this process is conducted at a low temperature of 120 °C, which substantially suppresses the generation of new defects associated with interfacial instability during high-temperature annealing, while the supercritical state enhances the nitridation effect. Therefore, SCN_2_O annealing presents a promising POA route for the realization of high-performance SiC power MOSFETs.

#### 2.2.2. Gate Oxide Doping

Beyond nitridation, modification of the gate oxide by introducing foreign species such as sodium (Na) [71], phosphorus (P) [72,73,74,75,76], boron (B) [77,78,79,80], and alkali/alkaline earth elements like barium (Ba) [81,82,83,84] has been explored for effective passivation of the SiO_2_/SiC interface and enhancement of *µ*_fe_.

**Figure 9:**
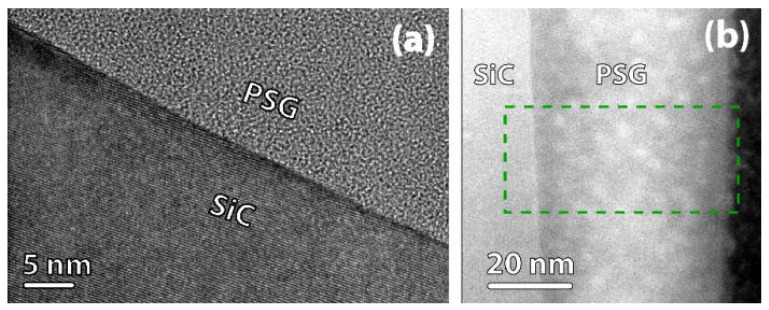
(**a**) HRTEM image of the SiC/PSG interface, showing atomic steps from long-range roughness. (**b**) HAADF-STEM (Z-contrast) image (contrast-enhanced) revealing bright spots in the PSG and a lower-mass dark layer at the interface [75].

Okamoto et al. incorporated P by high-temperature annealing of SiO_2_/4H-SiC (0001) capacitors in a POCl_3_ ambient. This process reduced the *D*_it_ near the conduction band edge to ~1 × 10^11^ eV^−1^·cm^−2^ and yielded lower NITs compared to nitridation techniques, resulting in a high *µ*_fe_ of 89 cm^2^·V^−1^·s^−1^ [72]. Alternatively, introducing P into SiC via ion implantation prior to thermal oxidation has also been proven effective in reducing *D*_it_ [73]. However, P incorporation tends to convert SiO_2_ into a phosphosilicate glass (PSG) layer, which can lead to greater *V*th instability under high-temperature and high-field stress [74]. Taillon et al. demonstrated through HRTEM and HAADF-STEM analysis that the SiC/PSG interface exhibits significant atomic-scale roughness, with the PSG layer maintaining a uniformly amorphous structure, as shown in Figure 9a. Moreover, they observed a non-uniform distribution of phosphorus throughout the PSG layer, which may underlie the observed performance-limiting polarization instabilities, as shown in Figure 9b [75].

Recent studies have also demonstrated promising results with antimony (Sb) [85,86] and arsenic (As) [86], and have attributed the mobility enhancement caused by these Group-V elements to a counter-doping effect. When these atoms accumulate at the SiO_2_/SiC interface, they act as donors by substituting for Si or C atoms. As a result, the inversion channel of the MOS is displaced deeper into the SiC bulk, thereby reducing Coulomb scattering, leading to an increase in *µ*_fe_, especially at low gate voltages where Coulomb constitutes the dominant mobility limiting factor. At high gate voltages, however, surface roughness scattering becomes predominant, and the benefit of counter-doping diminishes. The *µ*_fe_ versus gate voltage curves after doping with different Group-V elements exhibit a sharp peak followed by a marked decline in mobility [87]. Specifically, As and Sb doping yield peak *µ*_fe_ values of 160 and 100–120 cm^2^·V^−1^·s^−1^, respectively—which are higher than that obtained with P. Nevertheless, under high gate voltages, the mobilities of Sb- and As-doped devices fall significantly below that of the P-doped case. Therefore, for practical device applications, P counter-doping offers a more favorable trade-off.

In recent years, progress has also been made in the study of boron (B) doping in gate oxides. Okamoto et al. performed B diffusion through a pre-grown dry thermal oxide, resulting in reduced *D*_it_ near the conduction band edge of 4H-SiC. The fabricated 4H-SiC MOSFETs achieved a peak *µ*_fe_ of 102 cm^2^·V^−1^·s^−1^ [77]. Since B acts as an acceptor in SiC, this mobility enhancement cannot be attributed to counter-doping; rather, it stems primarily from structural modifications at the SiO_2_/SiC interface. According to earlier studies on POCl_3_ [76], interfacial stress can be alleviated by the incorporation of network-forming species. Specifically, due to the lower electronegativity of Si, B atoms preferentially occupy Si lattice sites instead of C sites. This substitution weakens the oxygen bond strength and promotes stress relaxation within the interfacial oxide layer [78], thereby raising the *µ*_fe_ peak. Moreover, B diffusion has been shown to effectively reduce NITs, contributing further to *µ*_fe_ improvement.

A notable strategy for incorporating B into gate oxides combines N_2_O oxynitridation with B diffusion [79,80]. Since carbon clusters and NITs constitute the dominant fraction of interface traps, the combined effect of B diffusion and oxynitridation is beneficial for optimizing SiO_2_/SiC interfacial properties. Compared to B doping alone, this hybrid method yields an average *µ*_fe_ peak of about 160 cm^2^·V^−1^·s^−1^ at low gate biases, with the mobility attenuating only to approximately 120 cm^2^·V^−1^·s^−1^ even when the gate voltage is raised to 30 V [87]. Other research groups have reported *µ*_fe_ peaks above 160 cm^2^· V^−1^·s^−1^ at low fields by combining B treatment with superficial Sb doping; however, the mobility degradation under high gate bias in such schemes is considerably more pronounced than in the B-plus-N_2_O case. It is also noteworthy that boron-doped oxides maintain a positive *V*th without compromising the critical breakdown field of the dielectric.

Instead of the relatively light elements mentioned above, the use of heavier alkali (Rb and Cs) and alkaline earth elements (Ca, Sr, and Ba) for modifying the SiO_2_/4H-SiC interfaces has also been investigated [81,82]. The process mainly consists of a very thin interface layer of alkali or alkaline earth materials deposition, followed by a CVD-grown SiO_2_ layer and a subsequent high-temperature POA in O_2_ or O_2_/N_2_ atmospheres.

Current studies show that thin layers of alkali metals (Rb and Cs) only raise the *µ*_fe_ to approximately 25 cm^2^·V^−1^·s^−1^ [87]. In contrast, alkaline earth elements Sr and Ba yield significantly higher peak mobilities of 40 and 85 cm^2^·V^−1^·s^−1^, respectively, with Ba also giving the lowest *D*_it_ (~3 × 10^11^ cm^−2^·eV^−1^ at *E*_C_ − *E =* 0.25 eV) [81]. While the exact enhancement mechanism is not fully understood, both elements lower *D*_it_ near the conduction band edge. Their ionic bonding (more flexible than covalent SiO_2_ bonding) likely passivates dangling bonds and alters the interfacial chemistry. Moreover, Sr and Ba can accelerate SiC oxidation (metal-enhanced oxidation, MEO), suggesting more efficient carbon removal. Ba also provides stable *V*th under bias temperature stress under gate bias conditions of 2 MV·cm^−1^ at 175 °C [81]. This behavior is consistent with the high chemical reactivity of Ba and its thermodynamic tendency to form compounds such as silicates.

However, Ba-based interfaces face challenges: non-uniform oxidation of the MEO reaction and degradation of insulating properties [83,84]. Non-uniform oxidation leads to oxide surface roughening and oxide thickness variations, which can be partially alleviated by employing thicker SiO_2_ capping layers and optimized process conditions [81,84]. More critically, in Ba-incorporated SiO_2_/SiC MOS, the leakage current rises sharply near 1.5 MV·cm^−1^ due to Fowler–Nordheim (FN) tunneling, and even with a SiO_2_ capping layer the breakdown field remains about half that of standard SiO_2_/SiC gate dielectrics [84]. Thus, Ba doping complicates gate dielectric design: Thicker oxides are required to ensure reliability, which partly offsets the performance gains achieved through interface optimization.

#### 2.2.3. Pretreament Before Thermal Oxidation

As previously discussed, SiC possesses the unique advantage of forming its native oxide, SiO_2_, via thermal oxidation. However, even prior to oxidation, the presence of initial surface defects on the SiC epitaxial layer—such as dangling bonds, C– or O– contaminants and adsorbates, and carbon-related point defects—also remains a primary cause of poor SiC/SiO_2_ interface quality [50,88,89]. To address this issue, surface pretreatment techniques, originally developed for Si technology and commonly employed before metallization or gate oxide formation, have been adapted for SiC. These methods serve not only to remove surface contaminants but also to weaken the robust Si-C bonds, thereby enhancing the quality of subsequently formed interfaces.

**Figure 10:**
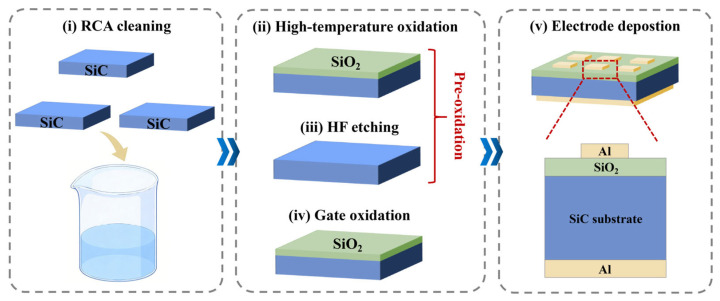
Schematic process flow for the fabrication of SiC MOS capacitors, emphasizing critical steps, including the “pre-oxidation” treatment, commonly referred to as the sacrificial oxidation process.

Several pretreatment methods prior to dielectric deposition for improving SiC surface characteristics have been reported, involving ultraviolet ozone treatment [90], hydrogen/nitrogen plasma processing [91,92], and direct atomic source nitridation treatment [93]. Early work by Afanas’ev et al. demonstrated the benefit of pre-oxidation ozone–UV cleaning, which effectively reduces the concentration of carbon clusters on the SiC surface and significantly improves surface quality [94]. Subsequent studies by Iwasaki et al. revealed that NH_3_ plasma pretreatment substantially lowers the *D*_it_, which is attributed to the nitrogen passivation of interface traps and the hydrogen termination of dangling bonds [95]. Nakashima et al. showed that nitrogen electron cyclotron resonance (ECR) plasma pretreatment not only markedly reduces NITs in the oxide but also effectively terminates Si-dangling bonds [96]. Recently, a sacrificial oxidation process has been reported: a thin sacrificial SiO_2_ layer is first grown on the SiC surface, removed by wet etching, and then regrown as the gate oxide by thermal oxidation, as illustrated in Figure 10 [97]. This approach smoothens the SiC surface without leaving significant carbon nano-islands, thereby effectively suppressing the generation of interface traps.

**Figure 11:**
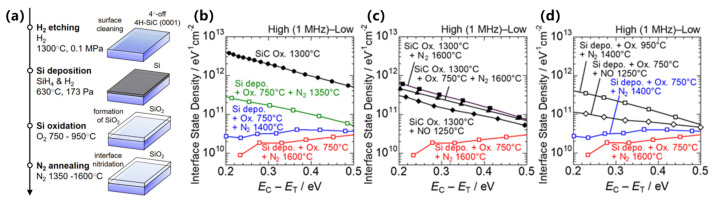
(**a**) Schematic process flow for fabricating SiC MOS capacitors via Si-on-SiC oxidation technology. Energy distribution of *D*_it_ measured by the high (1 MHz)–low method: (**b**) influence of N_2_ annealing temperature, (**c**) comparison between typical passivation methods (NO vs. N_2_ annealing), and (**d**) effects of oxidation temperature and post-oxidation treatment [98].

Even after surface pretreatment of SiC, direct oxidation inevitably introduces carbon-related defects [45,46,47], which degrade interfacial properties. Therefore, a cleaner oxidation approach has been proposed, referred to here as the Si-on-SiC oxidation process [98]. Its core idea is to produce a carbon-free SiO_2_ film and high-quality oxide/semiconductor interfaces without directly oxidizing SiC, thereby largely avoiding the interfacial degradation caused by carbon defects during SiC oxidation. As presented in Figure 11, the method comprises four steps: (i) in situ hydrogen (H_2_) etching of the SiC (0001) surface, (ii) deposition of a CVD-grown Si film, (iii) low-temperature (~750 °C) oxidation of the silicon layer to form SiO_2_, and (iv) high-temperature N_2_ annealing in the range of 1350–1600 °C instead of NO annealing to reconstruct the interface structure and passivate suboxide bonds and dangling bonds with N atoms. The entire process flow and conditions are carefully designed to ensure that only Si is oxidized while SiC remains intact. After optimization of the process parameters, the *D*_it_ measured by a high (1 MHz)–low method is on the order of 10^10^ cm^−2^·eV^−1^, representing two orders of magnitude reduction compared to that of an interface formed by direct SiC oxidation.

**Figure 12:**
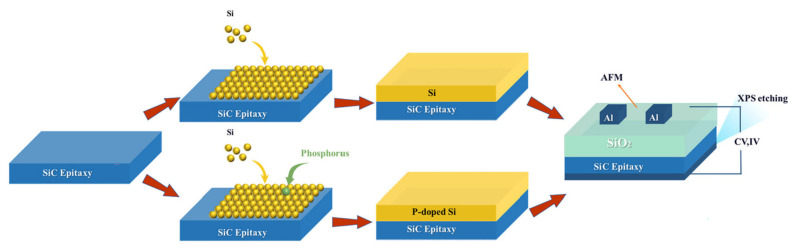
Schematic diagram illustrating the Si-on-SiC oxidation process with P doping [99].

More recently, Wang et al. further integrated this Si-on-SiC oxidation process with gate oxide doping [99]. Specifically, during the deposition of the Si layer on SiC, P atoms were introduced under precisely controlled doping concentrations to avoid excessive doping that would lead to PSG formation. Subsequent oxidation of the n-type Si layer yielded a carbon-free SiO_2_ film and a high-quality SiC/SiO_2_ interface, as shown in Figure 12. Compared to directly oxidized intrinsic Si, the phosphorus treatment reduced the *D*_it_ from 4.03 × 10^11^ cm^−2^·eV^−1^ to 2.01 × 10^11^ cm^−2^·eV^−1^. Moreover, the results indicated that P doping effectively suppressed flatband voltage hysteresis. Nevertheless, depositing high-quality Si layers on SiC remains challenging due to the significant lattice mismatch between SiC and Si. Most reported attempts have employed polycrystalline Si layers deposited by CVD, followed by gate oxide formation [100]. However, such approaches often result in a SiO_2_/Si/SiC trilayer structure because of excessive oxide thickness and partial oxidation. Therefore, further optimization of the fabrication process is required to achieve better outcomes.

Table 1 summarizes representative SiC surface pretreatment approaches reported in the literature and the corresponding key electrical metrics, including *D_it_* at E_C_-0.2 eV and dielectric breakdown field. Notably, all the reported data were extracted from MOS capacitor (MOSCAP) structures. To enhance cross-study comparability, the *D_it_* values listed in this table were consistently obtained using the high–low frequency capacitance method. The selected methods cover plasma pretreatment and irradiation using NH_3_ plasma, ECR-N plasma, and mixed N–H plasma, sacrificial oxidation of thin SiO_2_, and Si-on-SiC oxidation with subsequent treatments such as N_2_ annealing and dopant-assisted oxidation. Overall, these studies show that interface processing can reduce *D_it_* to the range of 10^10^–10^12^ eV^−1^·cm^−2^ while maintaining the breakdown field at approximately 9–11 MV·cm^−1^, providing a direct benchmark for comparing the effectiveness of different interface optimization routes.

## 3. High-k Gate Dielectrics for 4H-SiC MOS Devices: Interface Physics, Defect Engineering, and Material System

As SiC MOSFETs continue to evolve toward higher performance and higher reliability, thermally grown SiO_2_ gate dielectrics increasingly exhibit limitations in gate controllability and device reliability optimization. In this context, the introduction and application of high-k materials as gate dielectrics in SiC MOSFETs have become an important research direction. This approach offers new possibilities for achieving improved electrical performance and optimized gate structure design [101].

### 3.1. Interfacial Physics at High-k/SiC Interfaces and Defect-State Engineering

The effectiveness of high-k gate dielectrics in SiC MOSFETs largely depends on the interface quality with the SiC epitaxial layer. Unlike the relatively mature and stable interface in the Si and SiO_2_ system, the high-k and SiC interface is often in a thermodynamically non-equilibrium state. Interface physics involves complex chemical reactions, defect formation, and charge trapping processes. Therefore, a systematic understanding of the origin and evolution of defects at the high-k and SiC interface, together with the development of effective defect control strategies, is essential for achieving high-performance and high-reliability SiC MOSFETs.

**Figure 13:**
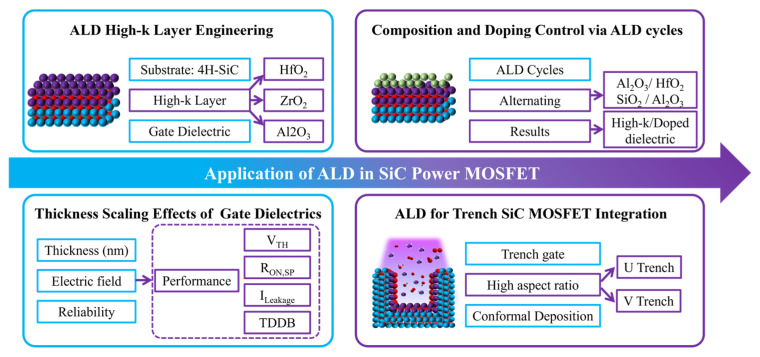
ALD Application for SiC Power MOSFET.

In SiC MOSFETs, high-k gate dielectrics are typically formed by thin film deposition techniques. Common methods include atomic layer deposition (ALD), chemical vapor deposition (CVD), and physical vapor deposition (PVD) [22,102,103,104]. Among these techniques, ALD is the most widely used approach for fabricating high-k gate dielectrics on SiC. This preference arises from atomic-scale thickness control, excellent film uniformity, and high process reproducibility [102]. CVD offers high deposition rates and mature process technology. However, the relatively high growth temperature can intensify interfacial reactions [103]. PVD features simple processing. Film density and interface quality strongly depend on subsequent annealing treatments [104]. The interface structures formed by these deposition methods are fundamentally different from those of thermally grown SiO_2_ and SiC interfaces. High-k materials cannot form a stable covalent network with SiC through thermodynamic oxidation. As a result, a non-ideal transition region is often present at the interface. This region may contain suboxides, incompletely bonded Si or C atoms, and a high density of structural defects. As SiC power MOSFETs continue to evolve toward lower on-state resistance and higher current density, trench-type SiC MOSFETs have emerged as an important device architecture for next-generation high-performance applications. This structure significantly reduces channel resistance. However, the thermal oxidation of SiC exhibits strong crystallographic orientation dependence. Under conventional thermal oxidation conditions, the oxidation rate of the Si-face (0001) at the trench bottom remains much lower than that of the a face (11–20) or the m face (1–100) at the trench sidewalls [105,106]. Under reverse bias conditions, the electric field is highly concentrated at the trench bottom region. A thinner bottom oxide layer therefore severely degrades breakdown characteristics and long-term gate oxide reliability. ALD provides excellent conformality for three-dimensional structures and atomic-scale thickness control. This technique is widely regarded as an effective approach for improving gate dielectric quality in trench-type SiC MOSFETs, particularly for achieving uniform oxide thickness in complex trench geometries, as shown in Figure 13 [105,106,107].

Extensive experimental and theoretical studies indicate that interface states at high-k and SiC interfaces mainly originate from carbon-related defects and suboxide structures. On the SiC surface, incomplete removal of C atoms during oxidation or surface cleaning can lead to the formation of carbon clusters, carbon interstitials, or carbon oxygen-related complex defects at the interface. These defects typically introduce deep levels or near conduction band states in the bandgap and significantly increase *D_it_*. In addition, partial reactions between high-k materials and the SiC surface may occur during deposition or annealing processes. These reactions can result in metastable bonding configurations such as Si–O–C or Si–O–High-k structures. Such suboxide-related structures disrupt interfacial chemical stability and introduce additional electrically active defects [108,109,110]. Therefore, from a material perspective, suppression of interface states is essentially achieved by controlling interfacial reaction pathways and defect formation mechanisms.

In addition to interface defects, bulk defects within high-k films also influence interfacial physical behavior through electric field coupling effects [111]. Typical bulk defects include oxygen vacancies, impurity atoms, and structurally disordered regions. Under high-electric-field or high-temperature conditions, these bulk defects act as charge trapping centers and interact with interface states. This interaction forms a complex trap network. The coupling between interface states and bulk defects leads to pronounced nonlinear defect behavior in high-k and SiC systems [112]. Interface states and bulk defects in high-k and SiC structures exhibit complex dynamic behavior under high-electric-field and high-temperature stress. Charge transport and trapping processes between interface states and bulk defects constitute the physical origin of reliability issues such as bias temperature instability, *V*th drift, and time-dependent dielectric breakdown [113]. From material and interface physics perspectives, the core requirement for high-k gate dielectrics in SiC MOSFETs is to minimize intrinsic defect density while maintaining adequate dielectric performance. In addition, the formation and evolution of interface defects must remain controllable and predictable. Achieving this goal relies on coordinated optimization of material selection, deposition processes, interface engineering, and post-deposition treatments.

### 3.2. HfO_2_-Based High-k Gate Dielectrics

Among existing high-k gate dielectric materials, HfO_2_ has been one of the earliest candidates introduced into SiC MOSFET research. This choice is attributed to a relatively high dielectric constant, good thermal stability, and extensive application experience in Si Complementary Metal-Oxide-Semiconductor (CMOS) technology [114,115,116]. In recent years, some studies have been carried out on interface properties, electrical performance, and reliability issues of HfO_2_-based gate dielectrics in SiC MOSFETs. Research outcomes in this area are highly representative within the field of high-k gate dielectrics.

#### 3.2.1. HfO_2_/SiC Interfacial Chemistry and Origins of Interface States

The dielectric constant of HfO_2_ typically ranges from 20 to 25, which is significantly higher than that of conventional SiO_2_. This property enables a smaller EOT while maintaining a relatively large physical thickness. As a result, gate leakage current is effectively reduced and gate controllability is enhanced [114,115]. In addition, HfO_2_ exhibits a high melting point, above 2700 °C, and relatively good thermal stability. These characteristics allow partial compatibility with the high-temperature processing environment required for SiC MOSFET fabrication [116]. However, subsequent studies have demonstrated that the HfO_2_ and SiC system exhibits significant differences in interface chemistry and defect behavior compared with the HfO_2_ and Si system. These differences have become key factors limiting the effectiveness of HfO_2_ gate dielectrics in SiC MOSFET applications.

#### 3.2.2. Electrical Characteristics and Reliability of Single-Layer HfO_2_ Gate Stacks

Although HfO_2_ shows promising potential as a high-k gate dielectric for 4H-SiC devices, the interface quality between HfO_2_ and 4H-SiC remains insufficient. Reported *D_it_* values are still relatively high and typically fall in the range of 10^12^ to 10^13^ eV^−1^·cm^−2^. This level of *D_it_* limits further improvement of device electrical performance. Many studies have shown that, when HfO_2_ is directly deposited on the SiC surface, the resulting *D_it_* is usually significantly higher than that of optimized SiO_2_ and SiC interfaces. This effect is particularly pronounced near the conduction band edge, where a high density of interface states is commonly observed [13,117,118]. From the perspectives of material properties and interface physics, this issue mainly originates from two factors. First, HfO_2_ exhibits strong oxidation capability. During deposition or subsequent annealing, non-equilibrium oxidation reactions can be induced at the SiC surface. These reactions lead to the formation of Si–O–C bonds or suboxide structures and strongly affect channel electron transport [117]. Second, the conduction band offset between HfO_2_ and SiC is only about 0.7 eV [119]. This relatively small barrier height facilitates electron injection and increases leakage current under operating electric fields. As a result, gate controllability and device reliability are further degraded [118,119,120]. Due to these limitations, the improvement in *µ*_fe_ remains limited even when HfO_2_ is employed as the gate dielectric. Studies by Sera Kwon et al. demonstrated that post-deposition nitridation annealing in NH_3_ can effectively suppress interface and bulk defects. This treatment improves the insulating properties and reliability of HfO_2_ and SiC gate dielectrics. However, *D_it_* remains on the order of 10^13^ eV^−1^·cm^−2^, which is still significantly higher than that of nitrided SiO_2_ and SiC interfaces [121].

From a reliability perspective, oxygen vacancies and deep-level defects inevitably present in HfO_2_ films readily participate in charge trapping processes under high-electric-field and high-temperature stress. These defects represent major physical origins of positive bias temperature instability and *V*th drift. Under the high-operating-voltage conditions of SiC MOSFETs, electron injection and trap-assisted transport effects become particularly pronounced and accelerate device aging. In addition, studies have shown that HfO_2_ may undergo local crystallization or phase transformation during high-temperature annealing. These processes reduce the effective dielectric breakdown field and can introduce new structural defects near the interface [121]. The combined effects of these factors indicate that a single HfO_2_ gate dielectric often struggles to simultaneously satisfy the requirements for high performance and high reliability in SiC MOSFET applications.

#### 3.2.3. Interface and Material Engineering of HfO_2_-Based Gate Stacks

**Figure 14:**
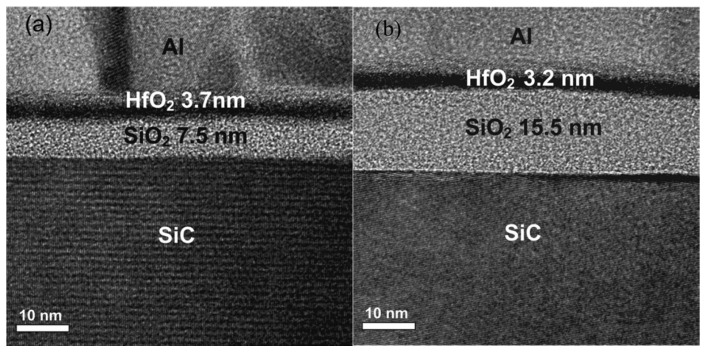
High-resolution TEM image of an HfO_2_ and SiC MOS structure with an inserted SiO_2_ interfacial layer (**a**) HfO_2_/SiO_2_ (7.5 nm) and (**b**) HfO_2_/SiO_2_ (15.5 nm) [124].

To address interface quality and reliability issues at the HfO_2_ and SiC interface, various modification strategies based on material and interface engineering have been proposed. Among these approaches, the introduction of an ultrathin SiO_2_ or SiON interfacial layer is widely regarded as one of the most effective methods [117,122]. Such an interfacial layer forms a relatively stable chemical structure on the SiC surface. This structure significantly reduces carbon-related interface defects and provides a high-quality template for subsequent HfO_2_ deposition. Mahapatra R. et al. introduced SiO_2_ and SiON interfacial layers into HfO_2_ and 4H-SiC gate stacks. The results showed that the SiON interfacial layer significantly reduced trapped charge density, leakage current, and flatband voltage shift without increasing *D_it_*. Device performance and reliability were therefore markedly improved [122,123]. Hsu and Hwu systematically investigated the influence of SiO_2_ interfacial layer thickness on the electrical characteristics of HfO_2_ and SiO_2_ and 4H-SiC gate structures [124]. The study demonstrated that, while an ultrathin SiO_2_ interfacial layer (~7.5 nm) effectively suppresses HfO_2_-induced C–V dispersion and improves interface properties, an excessively thick SiO_2_ layer (15.5 nm) causes carbon interstitial accumulation in the SiC substrate, leading to severe electrical degradation, thereby highlighting the critical importance of optimizing interfacial layer thickness in HfO_2_/SiC gate stacks, as shown in Figure 14.

To address the insufficient band offset when HfO_2_ is directly employed as the gate dielectric in SiC MOS structures, Mahapatra et al. systematically investigated the band alignment and electrical characteristics of HfO_2_ and SiO_2_ and SiC gate dielectric stacks [28]. The results showed that the valence band offset at the HfO_2_ and SiC interface is approximately 1.5 eV. After the insertion of an ultrathin SiO_2_ interfacial layer, a valence band offset of about 2.2 eV was formed at the SiO_2_ and SiC interface. This change effectively increased the carrier injection barrier. Correspondingly, the electron barrier height in the stacked structure increased to about 1.5 eV. Gate leakage current was significantly reduced. The breakdown electric field also increased from about 3.4 MV·cm^−1^ in the HfO_2_ and SiC structure to approximately 4.5 MV·cm^−1^ in the HfO_2_ and SiO_2_ and SiC structure. These results demonstrate that the reliability limitations of the single-layer HfO_2_ and SiC system can be effectively mitigated through the introduction of a SiO_2_ interfacial layer.

As SiC power MOSFETs continue to evolve toward higher power density and lower on-state loss, trench-type SiC MOSFETs have gradually become the mainstream device architecture. This trend is driven by higher channel density and lower specific on-resistance. Compared with planar structures, trench MOSFETs effectively reduce channel resistance per unit chip area through vertical channel design and enhance current driving capability [125,126,127]. However, the complex three-dimensional electric field distribution in trench structures, particularly at the trench bottom and corner regions, imposes more stringent requirements on the electrical performance and reliability of gate dielectrics [126,128]. Against this background, the introduction of high-k gate dielectrics has become an important research direction for gate structure optimization in trench SiC MOSFETs. Owing to a higher dielectric constant, high-k materials help enhance gate controllability while alleviating local electric field crowding. This capability provides new technological pathways for achieving high-performance and high-reliability trench SiC MOSFETs [129,130].

**Figure 15:**
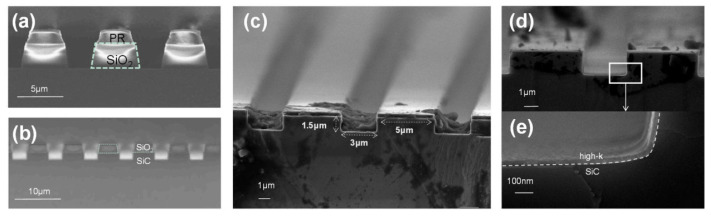
SEM images of 4H-SiC trench structures with conformal ALD-grown gate high-k dielectrics (**a**) after SiO_2_ etching, (**b**) after SiC etching, (**c**) the trench structure after BOE cleaning, (**d**) after ALD processing, and (**e**) magnification of the white-boxed area in (**d**) [117].

Recent studies have demonstrated that the introduction of high-k gate dielectrics into SiC trench structures plays a significant role in improving gate oxide reliability and suppressing leakage current. Huang et al. systematically investigated interface properties and electrical characteristics of ALD-grown HfO_2_ and SiO_2_ and HfO_2_ stacked gate dielectrics in 4H-SiC trench structures as shown in Figure 15 [117]. The results showed that a single HfO_2_ layer provides a high dielectric constant. However, due to the relatively small conduction band offset with SiC, a high gate leakage current is readily induced. In contrast, after inserting an ultrathin SiO_2_ interfacial layer between HfO_2_ and SiC, the breakdown electric field of the stacked gate dielectric increased from 4.1 MV·cm^−1^ to 6.5 MV·cm^−1^. At the same time, the gate leakage current was reduced by approximately one order of magnitude. This study further indicated that the SiO_2_ interfacial layer effectively modulates band alignment and suppresses tunneling conduction mechanisms under high-electric-field conditions. Dielectric stability was also improved in high-field regions such as trench corners. These results verify the strong potential of high-k and SiO_2_ stacked gate structures for application in SiC trench MOSFETs.

**Figure 16:**
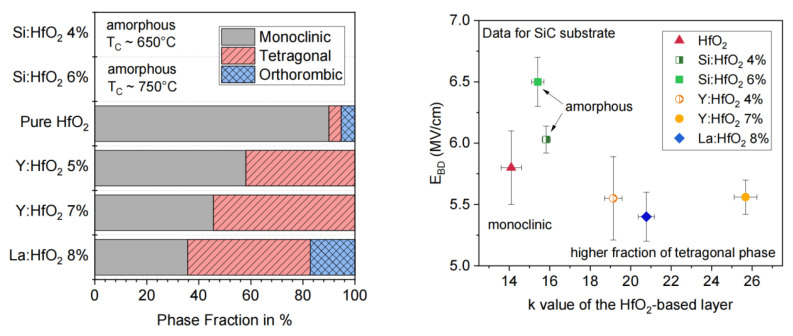
GIXRD characteristics and k-dependent E_BD_ modulation in Y-doped and La-doped HfO_2_ [27].

In addition, the formation of Hf-based composite oxides through material doping has been demonstrated to be an effective approach for reducing oxygen vacancy density, improving thermal stability, and optimizing interface and bulk defect behavior. Typical examples include HfAlO_x_, HfSiO_x_, and rare-earth-doped HfO_2_ [27,29,131,132]. Sandra Krause et al. systematically investigated the effects of dopant incorporation in HfO_2_ on the interface structure and electrical performance of SiC MOS devices. The results showed that Y and La doping increased the dielectric constant but degraded breakdown characteristics and leakage performance. In contrast, Si doping increased the crystallization temperature of HfO_2_ and maintained the film in an amorphous state. This behavior enabled lower leakage current and higher breakdown electric field on SiC, making Si-doped HfO_2_ the most suitable gate dielectric option for SiC power devices as shown in Figure 16 [27]. These interfacial and modified HfO_2-_based gate dielectrics generally exhibit improved *µ*_fe_, enhanced *V*th stability, and superior reliability characteristics in SiC MOSFETs. As a result, such material and interface engineering strategies have become a major focus of current research.

**Figure 17:**
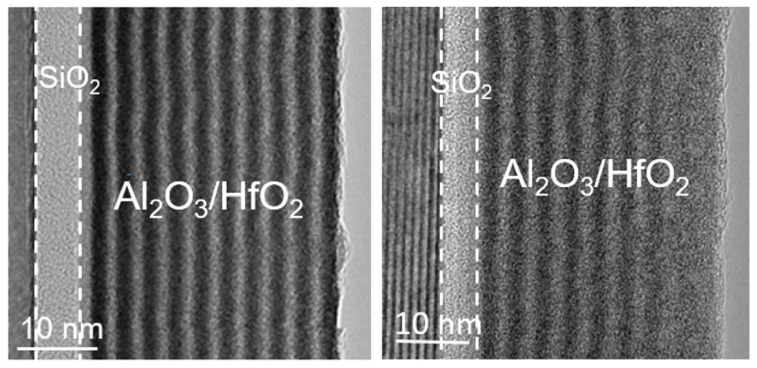
High-resolution TEM image of an HfO_2_ and SiC MOS structure with an inserted Al_2_O_3_ layer [24].

In HfO_2_ and SiC gate structures incorporating a SiO_2_ interfacial layer, interface states and leakage behavior can be partially improved. However, the low dielectric constant of SiO_2_ still limits electric field redistribution capability. Under this background, Lo Nigro et al. proposed Al_2_O_3_ and HfO_2_ nanolaminated high-k dielectric structures as an alternative solution and systematically investigated structural stability and electrical characteristics on 4H-SiC substrates [24]. The study showed that Al_2_O_3_/HfO_2_ nanolaminated dielectrics deposited by plasma-enhanced ALD effectively suppressed HfO_2_ crystallization while maintaining a high effective dielectric constant (~12–13) and good thermal stability, even after 800 °C annealing. Compared with single-layer HfO_2_, the nanolaminated structure exhibited reduced flatband voltage shift, lower trap density, and improved reliability, as confirmed by electrical measurements and HRTEM, as shown in Figure 17.

Overall, the high-k gate dielectric HfO_2_ exhibits clear advantages in EOT scaling and gate leakage current suppression in SiC MOSFETs. However, high *D_it_*, abundant bulk defects, and reliability concerns limit further improvement of *µ*_fe_ and long-term device stability. Through modification strategies such as interfacial layer insertion and material doping, the overall performance of HfO_2-_based gate dielectrics has been significantly improved. Nevertheless, the application of HfO_2_ in SiC MOSFETs still requires continued and in-depth investigation into intrinsic defect control and interface physical mechanisms.

### 3.3. ZrO_2_-Based High-k Gate Dielectrics

Beyond the extensively studied HfO_2_ gate dielectrics, ZrO_2_ has also attracted sustained attention as another group IV transition metal high-k oxide for SiC MOSFET gate dielectric applications [133]. ZrO_2_ has a crystal structure and dielectric constant comparable to those of HfO_2_, but differs in interfacial reactions, crystallization behavior, and band structure, leading to distinct advantages and challenges in SiC devices. Owing to its high dielectric constant and good thermal stability, ZrO_2_ is considered a promising high-k dielectric for improved gate oxide performance in SiC MOSFETs.

#### 3.3.1. Material Properties of ZrO_2_ and Interfacial Behavior on SiC

The dielectric constant of ZrO_2_ is comparable to that of HfO_2_ and typically ranges from 20 to 25. This value is significantly higher than that of conventional SiO_2_. Subsequent studies have shown that the ZrO_2_ and SiC interface also suffers from high *D_it_* and complex interfacial reactions. Interface defect characteristics and thermal stability behavior differ significantly from those of the ZrO_2_ and Si system. These factors limit the practical effectiveness of ZrO_2_ gate dielectrics in SiC MOSFET applications [30,134].

#### 3.3.2. Single-Layer ZrO_2_ Gate Dielectrics: Electrical Performance and Phase Stability

Similar to HfO_2_, the interface quality of ZrO_2_ and SiC remains a key factor limiting practical application. A stable native interface with low defect density is difficult to form between ZrO_2_ and SiC. As a result, directly deposited ZrO_2_ films usually exhibit high *D_it_* [134,135]. This behavior is particularly evident near the conduction band edge. Such a high *D_it_* significantly degrades electron mobility and *V*th stability in n-channel SiC MOSFETs.

In terms of processing, ZrO_2_ films are typically deposited by ALD [136]. ZrO_2_ readily crystallizes during high-temperature processing and device operation. The phase transition can introduce grain boundary-related defects. Grain boundaries can further degrade interfacial and bulk defect properties. Król et al. systematically studied the effect of ALD temperature (85–250 °C) on the growth of ZrO_2_ films on SiC. ZrO_2_ gradually changes from an amorphous phase to a tetragonal polycrystalline structure as the deposition temperature increases. The dielectric constant increases from 16 to 26. At the same time, the grain size increases and the number of grain boundaries increases. Post-deposition annealing also has a strong impact on the electrical performance of ZrO_2_. Proper annealing conditions can reduce bulk defects and improve the interface structure. Excessive annealing temperature can enhance crystallization and interfacial reactions. Electrical performance can degrade under such conditions [30,137]. Tedi et al. reported that ZrO_2_ transforms from the monoclinic phase to the tetragonal phase under annealing at 600–900 °C. The SiC/ZrO_2_ interfacial layer thickens from 2.5 nm to 10 nm. Annealing reduces the leakage current density by about one order of magnitude. The dielectric constant also increases significantly from 22 to 80. However, the breakdown voltage decreases and the interfacial charge increases. A clear trade-off exists between dielectric enhancement and reliability in ZrO_2_/SiC MOS structures [30]. Therefore, process optimization that balances defect suppression and thermal stability remains a key challenge for ZrO_2_ gate dielectrics.

#### 3.3.3. Interface Engineering and Materials Modification for ZrO_2_-Based Gate Stacks

To address these issues, interface engineering and stacked gate dielectric designs are widely adopted to improve the overall performance of ZrO_2_ gate dielectrics in SiC MOSFETs. Among them, inserting an ultrathin SiO_2_ interfacial layer between SiC and ZrO_2_ has been proven to be an effective strategy [26,138]. This configuration partially inherits the advantages of the SiC/SiO_2_ interface. It also leverages the high dielectric constant of ZrO_2_ to reduce the overall EOT. As a result, a balance can be achieved between interface quality and gate control. Wang et al. systematically studied the band alignment using first-principles calculations. They showed that the conduction band offset is only 0.45 eV when ZrO_2_ directly contacts 4H-SiC. This value is insufficient to suppress electron injection. After introducing a SiO_2_ interlayer, the conduction band offset of the ZrO_2_/SiO_2_/SiC stack increases significantly to 1.7 eV. Meanwhile, the interface remains free of in-gap states. The stable Si–O bonding and enhanced interfacial polarization in SiO_2_ effectively modulate the band alignment. This effect markedly improves the leakage behavior and reliability of ZrO_2_/SiC MOS structures [26]. These results indicate that a properly optimized ZrO_2_/SiO_2_ stacked gate structure can achieve lower *D_it_*, improved *µ*_fe_, and more stable *V*th characteristics. On this basis, Huang et al. further introduced ALD-grown ZrO_2_ and SiO_2_/ZrO_2_ gate dielectrics into 4H-SiC trench MOS capacitor structures [139]. They demonstrated that ALD can still provide continuous and uniform dielectric coverage on high-aspect ratio trench sidewalls. With an ultrathin SiO_2_ interfacial layer, the trench MOS devices show a higher breakdown electric field and lower leakage current. *D_it_* is also significantly reduced. These findings confirm that SiO_2_/ZrO_2_ interface engineering is also effective in trench-type SiC MOS structures. It is beneficial for improving the gate dielectric reliability and electrical performance of trench SiC power devices.

**Figure 18:**
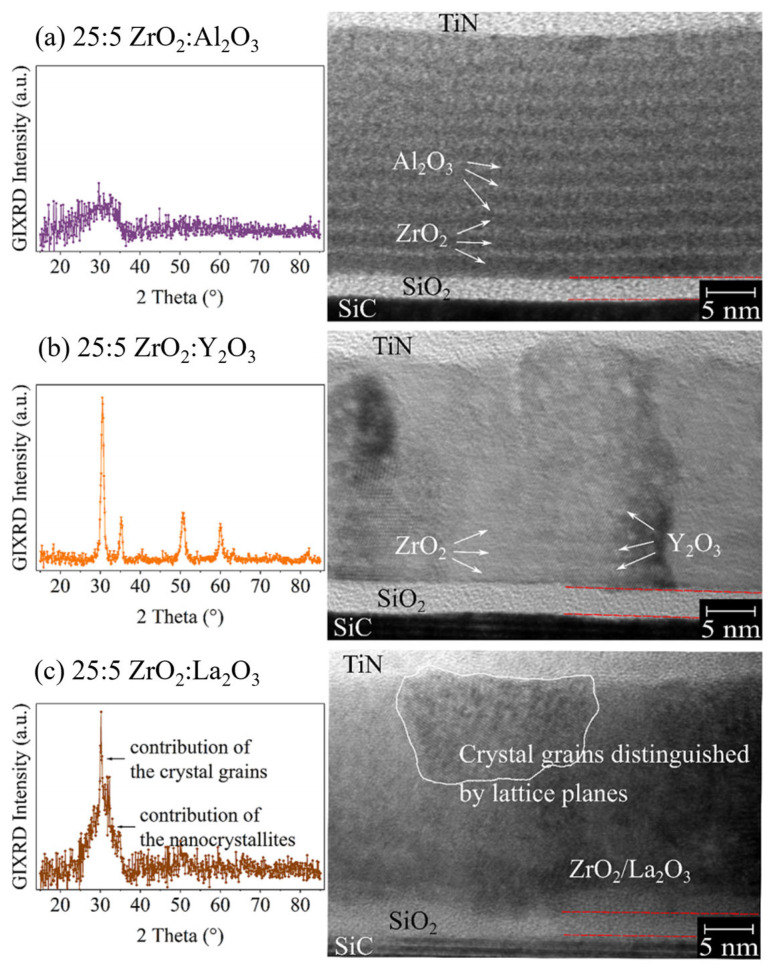
GIXRD patterns (**left**) and HRTEM images (**right**) of (**a**) ZrO_2_:Al_2_O_3_ (**b**) ZrO_2_:Y_2_O_3_ and (**c**) ZrO_2_:La_2_O_3_ nanolaminates with ALD cycle ratio 25:5, annealed at 500 °C for 1 min in Argon atmosphere [23].

Meanwhile, inserting an Al_2_O_3_ interlayer can also markedly improve the electrical performance and reliability. Sandra et al. investigated ZrO_2_-based multilayer dielectric structures for SiC power devices to reduce leakage and enhance reliability [23]. An ultrathin Al_2_O_3_, Y_2_O_3_, or La_2_O_3_ interlayer was inserted into ZrO_2_ films to form a nanolaminate structure. This design can block carrier transport and improve dielectric properties. The corresponding GIXRD and HRTEM results are shown in Figure 18. The Al_2_O_3_ interlayer provides the most pronounced improvement. The leakage current of thick ZrO_2_ films decreases by approximately two orders of magnitude. The breakdown electric field increases to 7.4 MV·cm^−1^. The crystallization temperature increases from 350 °C for pure ZrO_2_ to 750 °C, which helps maintain an amorphous structure. The effective dielectric constant is 13, which is significantly higher than that of pure Al_2_O_3_. In contrast, Y_2_O_3_ and La_2_O_3_ interlayers show limited effects on crystallization suppression and leakage reduction. Atomic diffusion and defect generation are considered the main reasons. Further optimization of the interface and deposition conditions reduces charge trapping by 50%. Overall, this study provides an effective route to achieve thick ZrO_2_ dielectrics with low leakage, high breakdown strength, and high-k characteristics. The proposed nanolaminate approach is promising for next-generation SiC power devices.

From the perspectives of device performance and reliability, SiC MOSFETs employing ZrO_2_ and ZrO_2_-based stacked gate dielectrics show reduced EOT and favorable initial electrical characteristics in several studies. However, the long-term reliability under high-temperature and high-field stress remains insufficiently understood. Key concerns include bias temperature instability and gate dielectric breakdown behavior. These issues require further systematic investigation. In addition, differences between ZrO_2_ and HfO_2_ in interfacial reaction activity and thermal stability provide an important basis for material selection and gate dielectric engineering in future SiC MOS technologies.

Overall, ZrO_2_ is a promising high-k gate dielectric for SiC MOSFETs. It offers potential advantages in EOT scaling and enhanced gate control. However, several challenges remain, including *D_it_* reduction, film phase stability, and long-term reliability. These issues require careful interface engineering and process optimization. Stacked gate structures incorporating a SiO_2_ interfacial layer, together with systematic control of deposition and annealing conditions, are regarded as key research directions to further advance the application of ZrO_2_ gate dielectrics in SiC MOSFETs.

### 3.4. Al-Based Dielectrics and Al-Containing High-k Gate Stacks

In SiC MOSFET gate dielectric systems, Al-based materials mainly include Al_2_O_3_, AlN, and Al-centered composite or doped high-k dielectrics, such as AlON, HfAlO_x_, and ZrAlO_x_. Compared with transition metal oxides such as HfO_2_ and ZrO_2_, Al-based dielectrics are not primarily valued for an ultrahigh dielectric constant. The key advantages arise from a wide bandgap, large band offsets, low intrinsic leakage current, and excellent thermal stability [109,140,141]. Al-based dielectrics in SiC MOSFETs can be used either as the main gate dielectric to reduce leakage and improve reliability, or as an interfacial control layer in stacked gate structures. In combination with SiO_2_ and high-k dielectrics, they help optimize gate electric field, interface trap density, and device reliability [142,143,144].

#### 3.4.1. Al_2_O_3_ Gate Dielectrics: Band Alignment, Interface States, and Reliability

Al_2_O_3_ is one of the most systematically investigated Al-based gate dielectrics and is also among the most widely adopted on the SiC. Its bandgap is approximately 7 eV, and its dielectric constant typically ranges from 8 to 10. Although its dielectric constant is lower than that of HfO_2_ and ZrO_2_, the large conduction band offset of more than 2 eV between Al_2_O_3_ and 4H-SiC provides a distinct advantage in suppressing electron injection and reducing gate leakage current [145,146,147,148].

In terms of fabrication, Al_2_O_3_ is commonly grown by ALD. The ALD process provides excellent thickness control, high film density, and conformal coverage over three-dimensional features, enabling uniform deposition in both planar and trench SiC MOS structures [149]. Experimental results show that, compared with thermally grown SiO_2_, the ALD-Al_2_O_3_/SiC system typically exhibits lower initial gate leakage current and a higher breakdown electric field. However, because the Al_2_O_3_/SiC interface does not form a highly idealized covalent interface as in the Si/SiO_2_ system, *D_it_* generally remains on the order of 10^12^ eV^−1^·cm^−2^, with a pronounced distribution of interface states near the conduction band edge. Guo et al. systematically compared the gate dielectric characteristics of single-layer Al_2_O_3_ on 4H-SiC prepared by thermal ALD and PEALD [109]. Compared with conventional thermal ALD, PEALD increases precursor reactivity and reduces hydroxyl groups and bulk defects in the film, thereby partially improving the *D_it_* distribution and enhancing breakdown performance. Zeng et al. investigated the as-deposited ALD-Al_2_O_3_/4H-SiC heterostructure and found Type-I band alignment with a conduction band offset of about 1.89 eV, together with a low leakage current density of 10^−10^ A·cm^−2^ and a high breakdown electric field of 9.3 MV·cm^−1^, while *D_it_* still remained on the order of 10^12^ eV^−1^·cm^−2^ [146]. Even under optimized conditions, single-layer Al_2_O_3_/SiC still cannot effectively suppress *D_it_* to the level of SiO_2_/SiC, highlighting the need for further interface engineering and structural design.

From the perspective of interfacial physics, interface states at the Al_2_O_3_/SiC interface mainly originate from residual carbon-related defects, incompletely passivated Si-dangling bonds, and interfacial suboxide structures. Because the oxidizing ability of Al_2_O_3_ is relatively mild, the deposition process usually does not trigger strong non-equilibrium interfacial reactions as observed for HfO_2_ or ZrO_2_. As a result, defect formation is largely determined by SiC surface pretreatment and the interfacial chemical state during the initial stage of deposition. This feature makes Al_2_O_3_ a gate dielectric that is highly sensitive to interface engineering and also highly tunable. Suvanam et al. investigated the interfacial structure and electrical optimization of ALD-grown Al_2_O_3_ on 4H-SiC. By comparing two pre-deposition cleaning procedures and different N_2_O post-annealing temperatures, the study showed that high-temperature annealing forms a SiO_x_ interlayer at the interface and converts the interlayer into a stable interface close to SiO_2_ at 1100 °C. The sample processed with an additional surface treatment in weak RCA cleaning after the typical surface treatment and annealed at 1100 °C exhibited lower oxide charges, smaller flatband voltage, lower leakage current, and higher breakdown field, indicating that the synergistic effect of surface pretreatment and high-temperature post-annealing is crucial for improving the Al_2_O_3_/4H-SiC MOS interface quality [150]. Jayawardhena et al. systematically compared the effects of different surface treatments on the performance of ALD-Al_2_O_3_/4H-SiC MOSFETs. The study found that NO nitridation combined with H_2_ annealing significantly reduced trap density, increasing the peak *µ*_fe_ to 52 cm^2^·V^−1^·s^−1^, which is clearly higher than that of conventional nitrided SiO_2_ devices [151]. This result demonstrates that pre-deposition surface engineering is a key step for unlocking the device potential of Al_2_O_3_/SiC gate stacks, while gate leakage reliability still requires further improvement. Usman et al. systematically studied the influence of annealing on the interfacial structure and electrical characteristics of ALD-Al_2_O_3_/4H-SiC. High-temperature annealing at about 1100 °C in N_2_ or N_2_O formed a stable SiO_2_ layer of about 1 nm at the interface, which markedly reduced fixed charges and improved C–V behavior and leakage characteristics [152]. This finding indicates that controlled formation of an interfacial SiO_2_ layer is one of the key mechanisms for enhancing the electrical quality of Al_2_O_3_/SiC gate structures.

In single-layer Al_2_O_3_/SiC gate stacks, annealing is a critical step for regulating interfacial defects and dielectric reliability. Moderate annealing effectively reduces oxygen vacancies, residual –OH bonds, and near-interface slow traps in Al_2_O_3_, improves film densification, and suppresses charge trapping. Annealing also promotes the rearrangement and passivation of unstable bonding configurations at the interface, thereby reducing *D_it_* and enhancing dielectric breakdown characteristics. However, excessively high annealing temperature or overly strong energy input can induce structural disorder in Al_2_O_3_, increase metal-related bonding, or intensify interfacial reactions, which leads to higher defect density and degraded electrical performance. The key objective of annealing in the single-layer Al_2_O_3_/SiC system is to achieve a delicate balance between defect repair and structural stability, which drives the growing interest in low-thermal-budget processes and plasma-assisted annealing. You et al. applied microwave plasma annealing to ALD-grown Al_2_O_3_ on 4H-SiC and achieved interface modulation without introducing an interfacial SiO_2_ layer. *D_it_* was reduced to about 6 × 10^11^ cm^−2^·eV^−1^, accompanied by significantly improved breakdown performance and electrical stability [145]. The improvement was mainly attributed to the repair of Al–O bond defects by oxygen plasma and the removal of interfacial Al–O–H bonds, indicating that plasma-assisted annealing is an effective approach for improving the interface quality of single-layer Al_2_O_3_/SiC. Miao et al. systematically investigated the material and electrical responses of ALD-deposited and high-temperature-annealed Al_2_O_3_ on 4H-SiC under heavy-ion irradiation. The study showed that polycrystalline Al_2_O_3_ formed after annealing at 1100 °C exhibited better post-irradiation structural stability than the amorphous film, including smaller changes in surface roughness, a more stable refractive index, and reduced radiation-induced damage. At the device level, the annealed samples showed much smaller increases in *D_it_* and flatband voltage shift after irradiation, together with less degradation of breakdown field, indicating stronger tolerance to single-event effects. By correlating material structure, interfacial defects, and device reliability, this work demonstrated that annealing-induced crystallization is a key route for improving the radiation reliability of Al_2_O_3_/SiC MOS gate dielectrics [153].

#### 3.4.2. AlN Gate Dielectrics: Heterointerfaces and Blocking Capability

In addition to Al_2_O_3_, AlN has also been gradually introduced into SiC MOS gate dielectric research as a wide-bandgap compound semiconductor with strong covalent bonding. AlN has a bandgap of about 6.2 eV, high thermal conductivity, and excellent thermal stability. The dielectric constant is about 8 to 9, which is comparable to Al_2_O_3_, while the band structure and interface type show clear differences. Unlike oxide gate dielectrics, AlN on SiC forms a semiconductor-like heterojunction interface. Both theoretical and experimental studies show that the AlN/4H-SiC system can exhibit Type-I band alignment with large conduction band and valence band offsets, which effectively suppress carrier injection under high electric fields. This intrinsic advantage makes AlN promising for improving breakdown performance and reducing gate leakage current [154].

**Figure 19:**
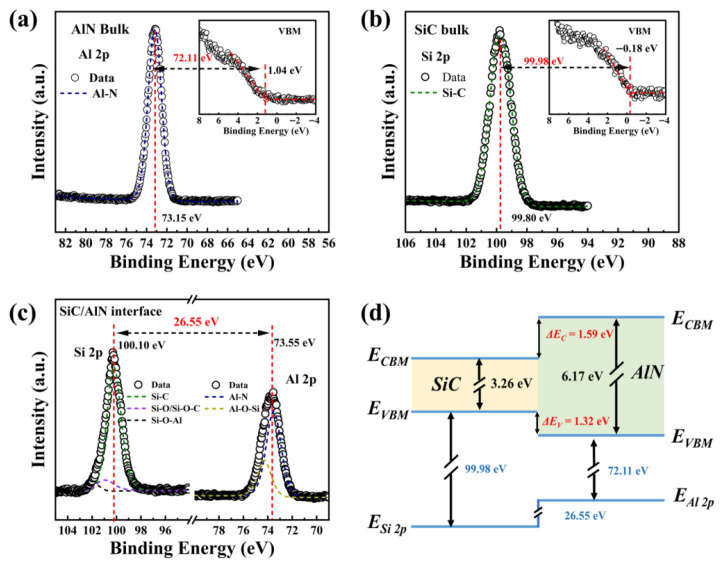
Type-I band alignment research of SiC and AlN grown by PEALD (**a**) Al 2p core level and valence band spectra of pure AlN. (**b**) Si 2p core level and valence band spectra of pure SiC. (**c**) Si 2p and Al 2p core levels at the SiC/AlN interface. (**d**) Band alignment of SiC and AlN [110].

In fabrication, AlN films are commonly grown by metal–organic chemical vapor deposition (MOCVD), PVD, or plasma-enhanced ALD. With proper optimization of deposition conditions and post-treatment processes, AlN/SiC MOS structures can exhibit low leakage current and good thermal stability [22]. However, because AlN is a strongly covalent material, interfacial defects mainly originate from nitrogen vacancies, interfacial mismatch stress, and structural disorder introduced during deposition, which limits further reduction in *D_it_*. Guo et al. deposited high-k AlN films on 4H-SiC by plasma-enhanced ALD and systematically studied the interfacial structure, band alignment, and electrical properties. The results showed Type-I band alignment with a conduction band offset of about 1.59 eV and a valence band offset of about 1.32 eV, together with relatively low *D_it_*, a breakdown field up to 11.4 MV·cm^−1^, and a large barrier height of 1.64 eV. These results indicate strong potential of AlN as a high-k gate dielectric for SiC MOS devices to enhance blocking capability as shown in Figure 19 [110]. However, the use of AlN alone can introduce relatively high leakage current [155]. Further systematic exploration is still required to fully leverage the intrinsic advantages of AlN for SiC power devices that demand high breakdown strength and high-temperature stability.

#### 3.4.3. Interface Engineering and Hybridization Strategies for Al-Based Gate Stacks

To fully exploit the advantages of Al-based dielectrics in SiC MOSFETs while mitigating the limitations in dielectric constant and *D_it_*, various modification strategies have been proposed based on interface engineering and material hybridization.

One of the most common approaches is to construct stacked gate structures such as Al_2_O_3_/SiO_2_, Al_2_O_3_/HfO_2_, or Al_2_O_3_/ZrO_2_. In these stacks, Al_2_O_3_ serves as an interfacial layer or barrier layer that suppresses oxygen vacancy diffusion in high-k dielectrics, reduces interfacial fixed charge density, and improves overall band alignment. The role of Al_2_O_3_ as an ultrathin interlayer or barrier in HfO_2_- or ZrO_2_-based gate stacks has been well demonstrated in the above-mentioned HfO_2_ and ZrO_2_ stacked structures by suppressing interfacial reactions and bulk defects and by improving band alignment and thermal stability [23,24]. Al_2_O_3_ can also act as an ultrathin interlayer to reduce *D_it_* at the SiO_2_/SiC interface and improve interface quality. Wang et al. introduced an ultrathin ALD-Al_2_O_3_ interlayer into an Al_2_O_3_/4H-SiC gate structure and formed a SiO_2_/Al_2_O_3_/SiC stack by Si deposition and reoxidation. The results showed that an Al_2_O_3_ thickness of about 1 nm reduced *D_it_* to about 3 × 10^11^ cm^−2^·eV^−1^ and simultaneously increased the breakdown electric field and improved flatband voltage stability, whereas further thickening of Al_2_O_3_ could degrade interface quality [156]. Building on this work, Ding et al. systematically investigated the synergy between a pre-deposited ultrathin Al_2_O_3_ interlayer and post-annealing. An Al_2_O_3_ thickness of about 1 nm combined with annealing at 900 °C effectively reduced the fixed charge density at the SiO_2_/SiC interface to about 4× 10^11^ cm^−2^ and reduced the near-interface trap density to about 2.5 × 10^11^ cm^−2^ while maintaining low leakage. In contrast, high-temperature annealing at 1200 °C induced Al diffusion and increased leakage current [140]. Therefore, introducing an ultrathin Al_2_O_3_ interlayer into SiO_2_/SiC gate stacks can effectively suppress interfacial defects and improve electrical stability, but the benefit depends on a strictly controlled annealing temperature window, since excessive thermal budget and the resulting Al diffusion can degrade dielectric reliability.

Thin SiO_2_/Al_2_O_3_ gate stacks also deliver favorable device performance. Zhai et al. proposed a low-oxygen-partial-pressure high-temperature oxidation process before Al_2_O_3_ deposition to preform an ultrathin SiO_2_ interfacial layer and construct a SiO_2_/Al_2_O_3_/4H-SiC gate stack. This approach formed a high-quality SiO_2_ interfacial layer with a thickness of about 0.4 nm, significantly reduced the SiC_x_O_y_ transition layer content, and lowered *D_it_* to 6.6 × 10^12^ cm^−2^·eV^−1^ at *E*_C_ − *E* = 0.2 eV, together with enhanced breakdown electric field and improved electrical stability [143]. This work demonstrates that precise control of the thickness and composition of the SiO_2_ interfacial layer is essential for exploiting the advantages of SiO_2_/Al_2_O_3_ stacks. Arith et al. introduced an ultrathin SiO_2_ interfacial layer combined with an ALD-Al_2_O_3_ gate dielectric in 4H-SiC MOSFETs and achieved a peak *µ*_fe_ of 125 cm^2^·V·^−1^·s^−1^ with stable behavior over a wide gate voltage range, along with a significant improvement in current drive capability [157]. This result indicates that a thin SiO_2_ interfacial layer effectively reduces interfacial defects, while Al_2_O_3_ provides high gate capacitance, making this stack a key structural design for high-mobility enhancement-mode SiC MOSFETs. Using a thermally grown ultrathin SiO_2_ interfacial layer combined with an ALD-Al_2_O_3_ gate stack, Arith et al. further demonstrated enhancement-mode 4H-SiC MOSFETs with a peak *µ*_fe_ of 125 cm^2^·V^−1^·s^−1^ and a subthreshold swing of 130 mV·dec^−1^, while maintaining enhancement-mode operation and a high on–off ratio at 300 °C [158]. Urresti et al. introduced a 0.7 nm ultrathin thermally grown SiO_2_ interfacial layer on 4H-SiC followed by an ALD-Al_2_O_3_ gate dielectric. *D_it_* was reduced to about 10^11^ cm^−2^·eV^−1^, enabling enhancement-mode SiC MOSFETs with a peak *µ*_fe_ of 265 cm^2^·V^−1^·s^−1^ [159]. Temperature-dependent characteristics showed that this gate stack effectively suppresses Coulomb scattering and shifts carrier transport to phonon-limited behavior, highlighting the strong advantage of SiO_2_/Al_2_O_3_ gate stacks in enhancing the *µ*_fe_ of SiC MOSFETs. Kil et al. introduced an ultrathin Al_2_O_3_ layer on nitrided SiO_2_/SiC gate structures and formed a stable interfacial dipole layer at the Al_2_O_3_/SiO_2_ interface. This dipole induced a clear positive shift in flatband voltage and device transfer characteristics, while *µ*_fe_ remained almost unchanged [160]. This work demonstrates that the Al_2_O_3_/SiO_2_ interfacial dipole effect enables *V*th tuning of SiC MOSFETs without degrading interface quality, providing an effective strategy for gate dielectric engineering.

Another important strategy is the introduction of multicomponent dielectrics such as AlON or Al–Si–O–N. Incorporation of a moderate amount of nitrogen into Al_2_O_3_ further suppresses oxygen vacancies and interfacial defects without significantly sacrificing the dielectric constant, while thermal stability is also improved. Takeuchi et al. systematically investigated the effects of nitrogen content and bonding configuration in PEALD-grown AlON films on the electrical characteristics of 4H-SiC MOS structures. With increasing nitrogen content, the fractions of Al–N and Al–O–N bonds increased, whereas Al–NO_2_ bonds were markedly reduced. As a result, the density of negative charge traps in the AlON layer and the low-field leakage current were significantly decreased [161]. The study demonstrated that precise control of nitrogen bonding states in AlON, rather than a simple increase in nitrogen concentration, is the key to achieving low-defect and highly reliable AlON/SiC gate dielectrics. Chen et al. proposed a low-temperature one-step PEALD process at 185 °C, in which NH_3_ and O_2_ were simultaneously introduced within a single ALD cycle to realize uniform nitrogen incorporation in AlON films. This approach effectively avoided the non-uniform doping issues commonly observed in conventional post-oxidation or post-nitridation processes [162]. The process enabled precise control of AlON composition and thickness and produced films with lower surface roughness, lower leakage current, and higher breakdown electric field, demonstrating the clear process advantage of PEALD for high-quality AlON gate dielectrics. Based on this process, Bai et al. fabricated AlON films by PEALD for 4H-SiC MOS capacitors and achieved Type-I band alignment. *D_it_* near the conduction band edge was reduced to about 7.6 × 10^11^ cm^−2^·eV^−1^, while the breakdown electric field reached 10.4 MV·cm^−1^. These results indicate that AlON achieves a superior balance between interface quality and dielectric reliability by combining the low-defect interface characteristics of Al_2_O_3_ with the high-breakdown advantage of AlN [108].

**Figure 20:**
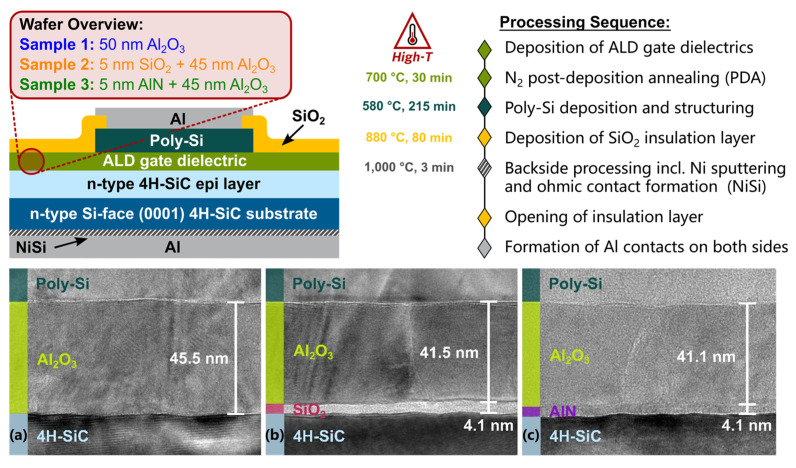
Process flow of Al_2_O_3_-based MOS capacitors and TEM overview images of (**a**) sample 1 (Al_2_O_3_/4H-SiC), (**b**) sample 2 (Al_2_O_3_/SiO_2_/4H-SiC) and (**c**) sample 3 (Al_2_O_3_/AlN/4H-SiC) [163].

However, Al_2_O_3_ faces reliability challenges in device integration. Ziegler et al. systematically investigated the electrical and structural properties of polysilicon-gated Al_2_O_3_-based high-k gate dielectrics under a thermal budget close to that of practical SiC MOSFET processing at about 1000 °C. Al_2_O_3_ maintained uniform breakdown behavior, low C–V hysteresis, and near-ideal flatband voltage after high-temperature processing. Nevertheless, time-dependent dielectric breakdown measurements revealed an intrinsic lifetime that was significantly shorter than that of commercial SiO_2_, mainly due to high-temperature-induced crystallization of Al_2_O_3_ and the formation of polycrystalline defects as shown in Figure 20 [163].

Overall, the value of Al-based gate dielectrics in SiC MOSFETs does not lie in pursuing a higher dielectric constant alone, but in the unique advantages in band engineering, defect suppression, and reliability enhancement. Through synergistic design with conventional SiO_2_ or other high-k oxides, Al-based dielectrics have become an indispensable component for realizing high-performance and high-reliability SiC MOSFET gate stacks.

### 3.5. Emerging High-k Gate Dielectrics: Rare-Earth Oxides and Multicomponent Stacks

Beyond conventional high-k dielectric systems, rare-earth doping and rare-earth-oxide composite gate dielectrics have gradually emerged as important approaches for further optimization of SiC MOS interfaces and reliability. Compared with single high-k oxides, rare-earth elements such as Sm, Y, La, Gd, and Ho feature large ionic radii and strong oxygen affinity, which suppress oxygen vacancy formation, regulate phase stability, and improve band alignment, thereby effectively reducing leakage current and enhancing breakdown performance.

Tarek et al. investigated the electrical performance of Sm_2_O_3_/ZrO_2_ bilayer gate dielectrics in 4H-SiC MOS structures and focused on the regulation of interface and dielectric quality under oxidizing and nitriding atmospheres. The results showed that the Sm_2_O_3_/ZrO_2_ stack formed under 50 percent O_2_ and 50 percent N_2_O exhibited the best performance, featuring a large conduction band offset of about 2.59 eV, a high-k value of about 28.8, and a high breakdown electric field of about 9.8 MV·cm^−1^ with a leakage current density of about 10^−6^ A·cm^−2^. Nitrogen incorporation effectively suppressed oxygen vacancies and reduced interface and bulk defect densities, which significantly lowered leakage current and enhanced breakdown performance [164]. These results indicate that synergistic optimization using rare-earth oxide and ZrO_2_ composite gate stacks together with nitridation can simultaneously improve the band alignment, dielectric properties, and reliability of ZrO_2_/SiC MOS structures.

Tarek et al. further combined moderate nitridation with a bilayer structure composed of the rare-earth oxide Ho_2_O_3_ and the transition metal oxide ZrO_2_, and demonstrated synergistic improvement in high-k/SiC interface quality and band alignment [165]. The study showed that oxygen vacancy suppression and effective nitrogen passivation were achieved simultaneously, leading to the formation of more stable Zr–O, Ho–O, and Zr–O–Si and Ho–O–Si bonding configurations, while preventing excessive growth of undesirable SiO_2_ at the interface.

Huang et al. systematically investigated the device-level performance of Al_2_O_3_/LaAlO_3_/SiO_2_ gate stacks in SiC power MOSFETs. The results showed that the introduction of the LaAlO_3_ high-k layer significantly increased gate capacitance, effectively suppressed *V*th shift at the same on-state resistance, and extended short-circuit withstand time to 1.55 times that of SiO_2_ gate structures. Further time-dependent dielectric breakdown analysis demonstrated a ten-year lifetime at an effective electric field of 4.8 MV·cm^−1^, indicating that La-based high-k dielectrics provide clear device-level advantages in achieving both low conduction loss and high reliability [166,167].

In summary, rare-earth oxide composite gate stacks combined with nitridation exhibit high dielectric constant, large conduction band offset, and excellent breakdown characteristics, offering clear advantages in suppressing oxygen vacancies and improving the high-k/SiC interface. However, because the number of reported studies remains limited, systematic evaluation is still required on reliability, thermal stability, and the evolution of interfaces and defects under high-temperature processing and long-term electrical stress to validate the engineering feasibility of these stacks as gate dielectrics for SiC MOSFETs.

### 3.6. Summary and Outlook of High-k Gate Dielectrics for SiC MOSFET

Overall, high-k gate dielectrics provide an important route to overcome the inherent limitations of conventional SiO_2_/SiC gate stacks in gate controllability, electric field distribution, and reliability. By introducing high-k materials such as HfO_2_, ZrO_2_, Al_2_O_3_, AlN, AlON, and rare-earth oxides, EOT can be effectively reduced while maintaining a relatively large physical thickness. This approach strengthens gate electric field control and reduces gate oxide electric field stress, with pronounced advantages in high-voltage and trench SiC MOSFETs. However, extensive studies show that the effectiveness of high-k materials in SiC MOSFETs does not depend solely on dielectric constant. *D_it_*, bulk defects, band alignment, and thermal stability often become the key factors that limit device performance and reliability. Table 2 summarizes representative high-k gate dielectric materials reported for SiC MOS devices. Notably, all the values collected in this table were extracted from MOSCAP structures. To improve cross-study comparability and minimize methodological discrepancies, the *D_it_* values at *E_C_*-0.2 eV reported in Table 2 were uniformly extracted using the high–low frequency method.

**Figure 21:**
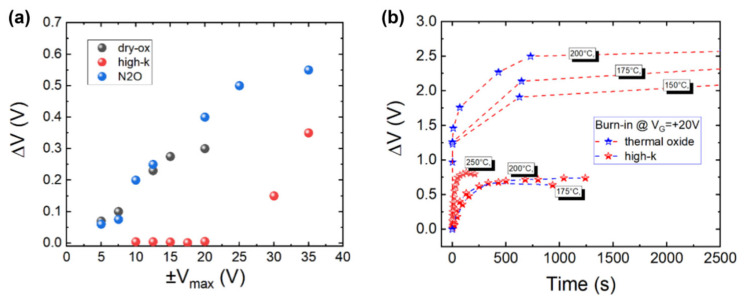
(**a**) Extracted hysteresis characteristics under sweep amplitudes from ±5 V to ±40 V for high-k and SiO_2_ dielectrics with and without post-oxidation nitridation. (**b**) Evolution of flatband voltage shift (ΔV) as a function of burn-in time at different temperatures for high-k and SiO_2_ gate dielectrics [168].

The selection of dielectric layers and interface treatment processes also plays a critical role in the electrical stability and reliability of SiC MOS devices [168]. As shown in Figure 21a, static hysteresis measurements reveal that N_2_O annealing does not effectively suppress charge trapping and detrapping at the SiO_2_/SiC interface, with pronounced hysteresis still observed. In contrast, the high-k dielectric exhibits virtually no hysteresis within a gate bias range of ≤20 V, demonstrating superior interface charge control and static *V*th stability. Furthermore, analysis of the flatband voltage shift (ΔV) as a function of burn-in time and temperature (Figure 21b) indicates that, although ΔV saturates more rapidly at higher temperatures for both dielectrics, the saturation voltage of the high-k stack is significantly lower than that of the conventional SiO_2_ structure. This confirms that the high-k dielectric offers stronger resistance to charge injection and more stable electrical behavior under high-temperature bias stress. In summary, high-k dielectrics outperform conventional oxidation and nitridation processes in suppressing interface hysteresis and enhancing high-temperature reliability, providing an effective technical route to significantly improve the long-term stability of SiC MOS devices.

Therefore, the engineering feasibility of high-k gate dielectrics in SiC MOSFETs strongly depends on interface engineering and stacked gate structure design. By introducing ultrathin SiO_2_ or Al_2_O_3_ interfacial layers, constructing multilayer or composite high-k gate stacks, and combining doping and defect control strategies, a better balance can be achieved among gate controllability, interface quality, and reliability. Despite their advantages, the application of stacked gate dielectric structures in SiC MOSFETs still involves several limitations. The introduction of additional dielectric interfaces may lead to extra fixed charges and trap states in the high-k layer or at the high-k/SiO_2_ interface, which can capture carriers, induce *V*th instability, and potentially degrade electrical stability at elevated temperatures, challenging long-term device operation [169]. Meanwhile, thermally grown SiO_2_ remains the most mature and widely adopted gate dielectric for SiC trench MOSFETs, while high-k dielectrics and their stacked structures are still mainly at the research stage but are regarded as promising candidates for future trench devices once reliability and interface issues are further resolved. Future research on high-k gate dielectrics will gradually shift from optimization of individual material parameters to systematic co-design of intrinsic defects, interface physics, and reliability evolution mechanisms, providing critical support for realizing high-mobility, low-loss, and high-reliability SiC power MOSFETs.

## 4. Conclusions and Prospects

Overall, the key challenges of the 4H-SiC MOS system can be attributed to the fact that oxidation or deposition processes inevitably introduce interfacial reactions and non-equilibrium defect networks during the formation of high-quality gate dielectric films on the SiC surface. Carbon-related defects, suboxide structures, and oxygen vacancies are particularly prominent, leading to a high density of traps and charge capture centers in the near-interface region. These defects simultaneously limit channel transport, *V*th stability, and dielectric reliability. Existing studies mainly follow two research directions. One direction is interface repair and film modification based on thermally grown SiO_2_. The other direction focuses on dielectric material and structural design using high-k dielectrics and stacked structures. The common objective of both approaches is to achieve controllable regulation of interface chemistry and film defects, ultimately enabling gate dielectrics that combine high dielectric performance, low *D_it_*, and robust stability under high-temperature and high-electric-field conditions.

### 4.1. Conclusions

This review systematically summarizes key progress in SiC MOS gate dielectric films and interface engineering, with emphasis on two major strategies. One strategy is the optimization of SiO_2_ systems, and the other is the development of high-k gate dielectrics and stacked films. The discussion focuses on film structure evolution, defect chemistry, and interface reaction mechanisms. The SiO_2_-based route remains the most mature in terms of processing. Strategies such as interface nitridation, gate oxide doping, and surface pretreatment have proven effective in passivating defects, reducing *D*_it_, and enhancing *µ*_fe_ in SiC MOSFETs. While significant progress has been achieved, challenges remain in the precise control of nitrogen concentration, ensuring the reliability of introduced dopants, and achieving more complete elimination of carbon contamination at the interface.

High-k films and stacked structures provide a broader material design space for reducing EOT and oxide electric field strength while enhancing gate control. However, when applied to SiC, these materials are more prone to non-equilibrium interfacial reactions, accumulation of oxygen vacancies and fixed charges, and insufficient phase stability. In addition, unfavorable band alignment can amplify carrier injection and trapping processes under high-temperature and high-electric-field conditions, thereby degrading dielectric reliability and device stability. Therefore, a key understanding has emerged that the performance bottleneck of SiC gate dielectrics is fundamentally a coupled issue involving near-interface defect networks and film structural stability. Future breakthroughs require a tighter closed loop linking material design, interface control, and reliability mechanisms.

### 4.2. Prospects

Research on SiC MOS gate dielectrics is expected to transition from experience-driven process optimization toward predictive design oriented to defects and structure. On the one hand, in situ or quasi in situ characterization combined with multiscale modeling is required to establish the generation, migration, and transformation mechanisms of carbon-related defects, suboxides, and oxygen vacancies. Quantitative correlations between defect spectra, charge trapping kinetics, and dielectric reliability should be developed to enable design-oriented and reproducible interface engineering. On the other hand, low-thermal-budget interface repair and atomic-scale reaction control are emerging as important trends, with the potential to suppress defect rearrangement and uncontrolled interfacial reactions induced by high-temperature steps.

For high-k and stacked dielectric systems, future efforts should focus on the synergistic optimization of phase stability, defect chemistry, and band engineering. Suppressing crystallization and oxygen vacancies, constructing low-defect interfacial layers, and increasing band offsets are critical to reducing carrier injection and trap activation. These material strategies must be validated through standardized long-term reliability evaluation under high-temperature and high-electric-field conditions to ensure engineering feasibility, with particular attention to *V*th stability, charge trapping dynamics, and degradation mechanisms such as bias temperature instability (BTI) and time-dependent dielectric breakdown (TDDB).

Overall, the core bottleneck of the 4H-SiC MOS gate dielectric system does not originate from a single material or a single process step, but from a systemic problem governed by the coupling between the near-interface defect network and the structural stability of the dielectric film. The process-mature route based on thermally grown SiO_2_ enables interface repair and film modification, and interface nitridation together with dielectric doping can reconstruct the interfacial bonding network and reduce defect activity, yet the improvement remains constrained by high-temperature defect regeneration, the introduction of fixed charges, and the limited capability to precisely control elemental distributions. Pretreatment is also considered a viable strategy, but the development of more thorough, carbon-free process technology is required. The route centered on high-k dielectrics and stacked structures provides a broader material space to reduce EOT, alleviate oxide electric field strength, and enhance gate control, but it is frequently accompanied by non-equilibrium interfacial reactions, the accumulation of oxygen vacancies and fixed charges, and insufficient phase stability. Unfavorable band alignment can further amplify carrier injection and trapping under high-temperature and high-electric-field conditions, thereby degrading reliability and device stability. Future breakthroughs require predictive design targeted at defects and structure, supported by low-thermal-budget processing and ALD-enabled atomic-scale reaction control to achieve controllable regulation of interface chemistry and defects. Closed-loop optimization should be established by integrating in situ characterization, multiscale modeling, and standardized long-term reliability evaluation under high-temperature and high-electric-field conditions. These efforts will ultimately enable advanced gate dielectric film systems that combine low *D_it_*, high reliability, strong thermal stability, and manufacturable process windows, providing critical material support for next-generation high-performance SiC power devices.

## Figures and Tables

**Table 1:** Interface state density and breakdown electric field of different interface treatment for SiO_2_/SiC MOS.

Method	*D_it_* (eV^−1^· cm^−2^)	MV·cm^−1^	Reference
NH_3_ plasma pretreatment	5 × 10^11^	-	[95]
Electron cyclotron resonance N plasma Irradiation	~3 × 10^11^	~9	[96]
N-H mixed plasma pretreatment	1 × 10^12^	10.86	[92]
Sacrificial oxidation of a thin SiO_2_ layer	6 × 10^11^	9.89	[97]
Si-on-SiC oxidation	7 × 10^11^	9.5	[99]
Si-on-SiC oxidation + N_2_ annealing	1.8 × 10^10^	9.8	[98]
P-doped Si-on-SiC oxidation	2 × 10^11^	9.0	[99]

**Table 2:** Summary of dielectric constant, breakdown electric field, and interface state density of high-k gate dielectrics for SiC MOS devices.

Structure	E_BR_ (MV·cm^−1^)	k Value	*D_it_* (eV^−1^·cm^−2^)	Reference
HfO_2_/SiC	-	20	2 × 10^13^	[14]
HfO_2_ (45 nm)/SiC	5.3	15	5 × 10^12^	[120]
HfO_2_ (127 nm)/SiO_2_ (8 nm)/SiC	6.6	14–15	-	[123]
HfO_2_ (29.8 nm)/SiO_2_ (10.7nm)/SiC	6.5	-	4.38 × 10^11^	[117]
ZrO_2_ (37.5 nm)/SiC	3.8	25	3 × 10^14^	[30]
ZrO_2_/Al_2_O_3_ (25:5 stacks)/SiC	7.4	13	-	[23]
ZrO_2_ (30.8 nm)/SiO_2_(10.7 nm)/SiC	5.8	-	3.2 × 10^11^	[139]
Al_2_O_3_ (39 nm)/SiC	9.3	-	1.44 × 10^12^	[146]
AlN (40.36nm)/SiC	11.4	-	7.83 × 10^11^	[110]
AlON (45.8 nm)/SiC	10.4	-	7.6 × 10^11^	[108]
Sm_2_O_3_ (4.8nm)/ZrO_2_ (3.6 nm)/SiC	9.8	28	-	[164]
Ho_2_O_3_ (2.6nm)/ZrO_2_ (3.3 nm)/SiC	11.05	26.4	-	[165]

## Data Availability

No new data were created or analyzed in this study.

## Abbreviations

The following abbreviations are used in this manuscript:

SiC	Silicon carbide
Si	Silicon
SiO_2_	Silicon dioxide
MOSFET	Metal-Oxide-Semiconductor Field-Effect Transistor
High-k	High dielectric constant
XPS	X-ray photoelectron spectroscopy
AES	Auger electron spectroscopy
EELS	Electron energy loss spectroscopy
*D_it_*	Interface state density
NITS	Near-interface oxide traps
*Q*_ox_	Fixed oxide charges
*µ*_fe_	Field-effect mobility
MOSCAP	MOS capacitor
EOT	Equivalent oxide thickness
POA	Post-oxidation annealing
FN	Fowler–Nordheim
ALD	Atomic layer deposition
CVD	Chemical vapor deposition
PVD	Physical vapor deposition
*V*th	Threshold voltage
BTI	Bias temperature instability
TDDB	Time-dependent dielectric breakdown
